# Repurposing DNA-binding agents as H-bonded organic semiconductors

**DOI:** 10.1038/s41467-019-12248-9

**Published:** 2019-09-16

**Authors:** Fengjiao Zhang, Vincent Lemaur, Wookjin Choi, Prapti Kafle, Shu Seki, Jérôme Cornil, David Beljonne, Ying Diao

**Affiliations:** 1 Department of Chemical and Biomolecular Engineering, University of Illinois at Urbana−Champaign, 600 South Mathews Avenue, Urbana, Illinois 61801 USA; 2 School of Chemical Sciences, University of Chinese Academy of Sciences, Beijing, 100049 P.R. China; 3 Laboratory for Chemistry of Novel Materials, University of Mons, Place du Parc, 20, B-7000 Mons, Belgium; 4 Department of Molecular Engineering, Graduate School of Engineering, Kyoto University, Nishikyo-ku, Kyoto, 615-8510 Japan; 5 Department of Chemical Engineering, Center for Advanced Soft Electronics, Pohang University of Science and Technology, Pohang, 37673 Korea

**Keywords:** Electronic materials, Electronic devices

## Abstract

Organic semiconductors are usually polycyclic aromatic hydrocarbons and their analogs containing heteroatom substitution. Bioinspired materials chemistry of organic electronics promises new charge transport mechanism and specific molecular recognition with biomolecules. We discover organic semiconductors from deoxyribonucleic acid topoisomerase inhibitors, featuring conjugated backbone decorated with hydrogen-bonding moieties distinct from common organic semiconductors. Using ellipticine as a model compound, we find that hydrogen bonds not only guide polymorph assembly, but are also critical to forming efficient charge transport pathways along π−conjugated planes when at a low dihedral angle by shortening the end-to-end distance of adjacent π planes. In the π−π stacking and hydrogen-bonding directions, the intrinsic, short-range hole mobilities reach as high as 6.5 cm^2^V^−1^s^−1^ and 4.2 cm^2^V^−1^s^−1^ measured by microwave conductivity, and the long-range apparent hole mobilities are up to 1.3 × 10^–3^ cm^2^V^−1^s^−1^ and 0.4 × 10^–3^ cm^2^V^−1^s^−1^ measured in field-effect transistors. We further demonstrate printed transistor devices and chemical sensors as potential applications.

## Abstract (web summary)

To unlock the potential of biological semiconductors for printed flexible electronics, experimental evidence that reveals the material’s charge transport mechanism is required. Here, the authors report the charge transport mechanism in hydrogen-bonded DNA topoisomerase inhibitors.

## Introduction

Since the inception of the field of organic electronics in 1970s, academic and industrial efforts have led to remarkable advancements of solution-processible, low-cost organic semiconductors^1,2^, with over six orders of magnitude improvement in charge carrier mobility thanks to rapid materials innovation^3^. Their solution printability, high (opto)electronic performance and improved ambient stability have enabled commercial applications such as flexible lighting and displays and transparent solar cells. Current molecular designs of high-performance organic semiconductors usually conform to polycyclic aromatic hydrocarbons and their analogs containing heteroatom substitution (commonly S, O, N, Se);^4^ electrons are delocalized over extended π-conjugated system, imparting charge mobility when subjected to the electric field. Interestingly, many biomolecules and naturally derived bioactive compounds are π-conjugated to enable short- and long-range charge transfer during biochemical conversion, to impart desired optical properties, or to facilitate interactions with other biomolecules. Well-known examples include DNA^5–7^, oligopeptide^8–12^, and natural dyes^13–16^, which have been investigated as biological conductors or semiconductors. Distinct from typical organic semiconductors, biological π-conjugated molecules frequently utilize hydrogen bonding to impart specific and strong multivalent interactions, which are further stabilized by π–π interactions. Hydrogen bonding not only guides intra- and intermolecular assembly, but can also directly or indirectly contribute to electron transfer (i.e. tunneling) and charge transport (i.e. hopping or band-like) in some cases. For instances, hydrogen bond^17,18^ was shown to critically facilitate biological electron transfer through proteins; the electron transfer rate when tunneling across a hydrogen bond can exceed that of a carbon–carbon single bond owing to delocalized electron densities^19,20^. In peptide semiconductors, hydrogen bond between carboxylic acid and amino groups may be important to forming the proton conduction channels in hydrated environment^11,21^. In pigment semiconductors, large bathochromic shift was often observed upon hydrogen bond-driven association. It was thus inferred that hydrogen bond can induce strong inter- or intra-molecular electronic coupling by enhancing resonance, electron delocalization or by planarizing the conjugated backbone^14^. In naphthalene-diimide (NDI) derivatives, it was shown that hydrogen bonding drove cooperative supramolecular assembly and thus led to effective delocalization of excited states^22,23^. Furthermore, long-range charge transport along H-bond has been theoretically predicted in diketopyrrolopyrrole and perylene diimide pigments^16,24,25^. Although many H-bonded organic semiconductors have been reported^13,15,22–26^, so far there is no direct experimental evidence that shows hydrogen bond is involved in long-range electron/hole transport in current carrying solid-state electronic devices. The challenges lie in decoupling the effect of H-bond from that of π–π stacking, also in fabricating electronic devices with well-defined H-bonding pathways over long range (hundred nanometer to microns), which requires highly aligned morphology. In the few examples that measured electrical conductivities/field-effect mobilities of biomolecular conductors/semiconductors, the current was too low at device-relevant length scales (from tens of nanometers to microns). DNA, for instance, becomes an electrical insulator when the channel length goes beyond 40 nm^7,27^. Furthermore, for all aforementioned biological semiconductors, their compatibility with solution printing for large-scale assembly and electronic device fabrication remains a challenge.

In this work, we discover that a group of anticancer plant alkaloids and their derivatives, known as DNA topoisomerase I and II inhibitors, can serve as a promising source for mining solution printable, high-performance organic semiconductors. These compounds interact with DNA via π−π stacking and hydrogen bonding, and thereby intercalate between the DNA base pairs or bind with topoisomerase–DNA complex to inhibit DNA replication. Required by such anticancer properties, these compounds often possess highly co-planar conjugated backbone decorated with hydrogen-bond donors and acceptors; both features are conducive to intermolecular electronic coupling for efficient charge transport in solid-state assemblies. Indeed, we measure high intrinsic field-effect mobilities in well aligned ellipticine crystalline thin films along both the π−π stacking and the hydrogen bonding directions, which is further supported by quantum-chemical calculations. Interestingly, charge transport along the hydrogen bonding direction is modulated by the H-bond dihedral angles, which we infer by comparing two polymorphs of ellipticine. These experimental findings are enabled by our ability to precisely control the crystal polymorphism and to attain high degree of domain alignment of ellipticine via meniscus-guide coating. We further elucidate that a low dihedral angle and a close distance of the H-bonding pair together with wavefunction delocalization over the H-bonding moieties act in concert to raise the electronic coupling between molecules along the H-bond direction. We further demonstrate application of this biological semiconductor in solution printed organic field-effect transistor (OFET) devices and chemical sensors.

Although postulated in studies of pigment semiconductors^13–15,25^, our report provides direct experimental evidence on long-range electron/hole transport along hydrogen-bonded molecular networks. Moreover, our work shows the potential of mining new classes of organic semiconductors from vast databases of therapeutic compounds. Majority of bioactive compounds explored as pharmaceuticals fail at various stages of drug development (including ellipticine studied in this work). Re-purposing these failed drug candidates as organic semiconductors is therefore low-cost and could potentially enable new mechanisms of charge transport, signal transduction, and bio-recognition, given their bioactive materials chemistries.

## Results

### Electronic structures of ***π***-conjugated DNA topoisomerase inhibitors

**Fig. 1:**
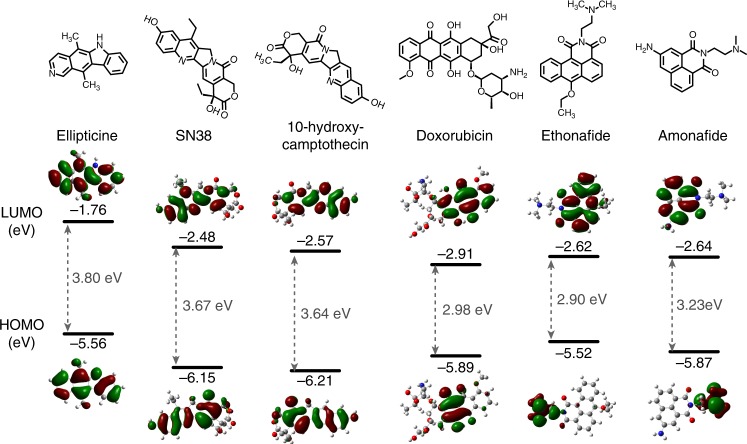
Molecular and electronic structures of DNA topoisomerase inhibitors tested. The top row shows the molecular structures of selected compounds possessing planar conjugated segments and hydrogen-bonding moieties (amine, amide, carbonyl, ester, ether, hydroxyl etc.) The bottom row shows corresponding optimized frontier orbitals and schematic energy level diagram of anticancer drugs as calculated using the B3LYP functional and 6–311 + G(d,p) basis set

For initial screening of promising organic semiconductors, we selected DNA topoisomerase inhibitors that exhibit π-conjugation, high co-planarity, and are rich in hydrogen bond donors and/or acceptors such as amine, amide, carbonyl, ester, ether, hydroxyl groups (Fig. 1). We calculated their electronic structure theoretically at the DFT(B3LYP)/6–311 + G(d,p) level. Corresponding highest occupied molecular orbital (HOMO), lowest unoccupied molecular orbital (LUMO) and bandgap (E_g_) are shown in Fig. 1. The theoretical calculations indicate that all six DNA topoisomerase inhibitors are wide-bandgap semiconductors, with E_g_ close to or exceeding 3 eV (for single molecules in vacuum). The experimentally measured optical bandgap in solution and in thin films ranged from 2.5 to 3.1 eV, which is substantially larger than typical organic semiconductors (Supplementary Fig. 1 and Supplementary Table 1). We note that organic semiconductors with wide bandgaps and uncompromised electronic performance have been pursued owing to their improved air stability, reduced photooxidation and light sensitivity, and high transparency to visible light^28,29^. Ellipticine, which is a plant alkaloid with anticancer properties, exhibits a calculated HOMO level in proximity to those of typical p-type organic semiconductors, making it most practical for charge injection from common metal electrodes among the six compounds evaluated. Therefore, we select ellipticine for further structure and charge transport property analysis.

### Crystal structure and calculated charge transport properties of ellipticine

**Fig. 2:**
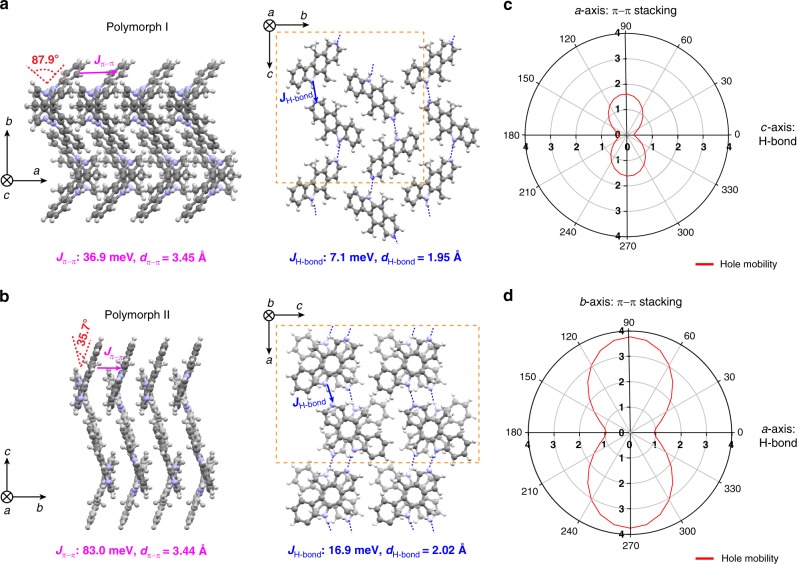
Crystal structures and calculated hole mobilities for ellipticine. **a**, **b** Crystal structures of **a** polymorph I and **b** polymorph II viewed from the π−π stacking and the H-bonding directions. The corresponding calculated charge transfer integrals and the measured stacking distances were labeled. The blue dash lines denote H-bonds (NH to N). The yellow dash lines enclose one unit cell of the crystal lattice. **c**, **d** Calculated hole mobilities (in cm^2^ V^−1^ s^ −1^) of ellipticine **c** polymorph I and **d** polymorph II. In both cases, 0° corresponds the H-bonding direction, and 90° the π−π stacking direction

In this section, we discuss the role of π–π stacking and H-bonding in guiding the assembly of ellipticine and the emergent charge transport properties predicted from quantum-chemical simulations on the basis of experimental crystal structures (see Methods). Using various single-crystal growth and solution printing techniques^30^, we obtained two different crystal polymorphs of ellipticine. Their single-crystal structures are shown in Fig. 2a, b, Supplementary Table 2 and Supplementary Fig. 2. The two polymorphs exhibit similar packing motifs, featuring co-facial π stacks and H-bonding linkages between adjacent π stacks. Guided by these H-bond linkages, the columns of π stacks are arranged in a zig-zag fashion for both polymorphs, but at distinct dihedral angles of 87.9° for polymorph I vs. 35.7° for polymorph II (Fig. 2a, b and Supplementary Fig. 3). We note that such packing motif does not fall under the categories commonly observed in organic semiconductor systems, such as 1D slip-stack, 2D brick-wall, or 2D herringbone packing motifs^31,32^. We attribute the distinction to the presence of H-bonding in the ellipticine system. Specifically, polymorph I exhibits π−π stacking along the *a* axis at a distance of 3.45 Å; the corresponding hole transfer integral *J*_π–π_ is calculated as 36.9 meV (Fig. 2a). In comparison, polymorph II’s π−π stacking occurs along the *b* axis at a comparable distance of 3.44 Å, with a much higher *J*_π–π_ value of 83.0 meV associated with improved wavefunction overlap relative to polymorph I (Supplementary Fig. 2). Such a high hole transfer integral is on par with or even exceeds those of high-performance organic semiconductors such as C12-BTBT isomers^33^ and TIPS-pentacene^34^. Orthogonal to the π−π stacking direction is the H-bonding direction, which is along the *c*- and *a* axis for polymorph I and II, respectively. Again, the H-bond lengths are closely matched (1.95 Å and 2.02 Å for I and II, respectively) but the hole transfer integral is substantially higher for polymorph II, with *J*_H bond_ of 16.9 meV vs. 7.1 meV for polymorph I. This difference is attributed to a lower dihedral angle between the molecular planes of the H-bonding pair for polymorph II (35.7° vs. 87.9° for polymorph I). The hole transfer integrals for other molecular pairs are summarized in Supplementary Fig. 4, which are relatively low given large intermolecular distances and/or small overlap. Altogether, we expect higher hole mobility for ellipticine polymorph II, with non-negligible contribution from the H-bond direction.

Besides charge transfer integrals, we further calculated reorganization energies and predicted the hole mobilities in the hopping limit (Fig. 2c, d and Supplementary Fig. 5). The electron transfer integrals and electron mobility distributions are also computed (Supplementary Fig. 4 and 6), however not discussed owing to the practical challenge of electron injection for charge transport property measurements of ellipticine. With a calculated hole reorganization energy of 128 meV, we obtained higher hole mobilities for polymorph II owing to its higher charge transfer integrals. Both polymorphs exhibit anisotropic hole mobility distributions, with the highest mobilities appearing along the π−π stacking direction, and lower but considerable hole mobilities along the H-bonding direction. Notably, the calculated hole mobilities for polymorph II are as high as 1.0 cm^2^ V^−1^ s^−1^ and 3.7 cm^2^ V^−1^ s^−1^ along the H-bonding and the π−π stacking directions, reaching the range of high-performance organic semiconductors despite limited π conjugation of ellipticine. The predicted high mobility along the H-bonding direction suggests the important role of H-bonds in contributing to charge transport, which is rarely considered for the molecular design of organic semiconductors.

**Fig. 3:**
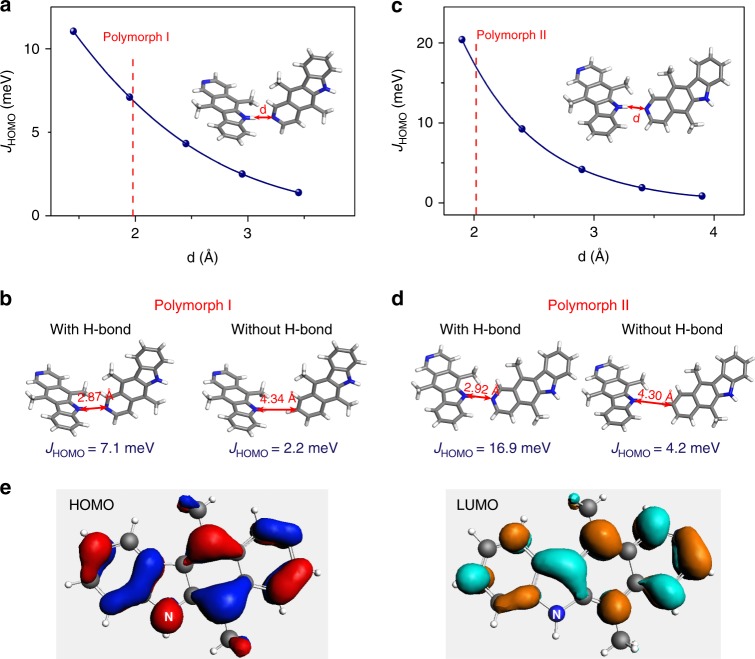
Theoretical calculations on the role of H-bond on electronic coupling. **a**, **c** Calculated hole transfer integral along the H-bonding direction as a function of NH…N distance for ellipticine with polymorph I and II. See Methods section for details. **b**, **d** Comparison of hole transfer integrals with and without H-bond for two polymorphs. The molecular pair without H-bond was created by replacing N with CH. Removal of H-bond requires adjustment of intermolecular distance from the original ~ 2.9 Å (N to N) to 4.3 Å (N to C) so that the two hydrogen atoms are separated by the sum of their van der Waals radii. We note that an intermolecular distance of 2.87 Å and 2.92 Å (N to N) corresponds to a H-bond length of 1.95 Å and 2.02 Å (N to H) in **a** for polymorph I and II, respectively. **e** Frontier molecular orbital topologies of ellipticine comparing the contribution of NH to HOMO vs. LUMO. The nitrogen atoms in the NH bonds are labeled in the HOMO and LUMO to facilitate direct comparison. Please note that this is a single molecule calculation result

An intriguing question is how H-bond contributes to the calculated charge transfer integral and charge carrier mobilities along the H-bonding direction. Do H-bonds directly participate in or indirectly facilitate electronic wavefunction overlap? The answer is both as we detail in the analyses below. We first investigated how the H-bonding strength and the bond length impact the hole transfer integral, and compared with the case without the H-bond by replacing N with CH for both polymorphs (Fig. 3 and Supplementary Fig. 7). We found that varying the H-bonding strength while keeping the bond length does not significantly impact the charge transfer integral along both the H-bonding and π-stacking directions (Supplementary Fig. 7 and 8). On the other hand, increasing the bond length of NH…N causes rapid drop in charge transfer integral for both polymorphs (Fig. 3a, c). This seems to suggest that the role of the H-bond is to bring closer the molecular pairs engaged in the H-bond, as to indirectly facilitate overlap of the electronic wavefunction of the conjugated molecular backbone. To validate this hypothesis, we removed the H-bond by replacing N with CH on one molecule of a dimer pair (Fig. 3b, d). For polymorph II, this change mandates an increase of the intermolecular distance from the original 2.92 Å (N–N) to 4.30 Å (N–C) and thus reduces the charge transfer integral from 16.9 to 4.2 meV; restoring the H-bond at this large distance of 4.30 Å did not improve the charge transfer integral. Same conclusion is obtained for polymorph I. This result suggests that H-bond indirectly contribute to electronic coupling by reducing the intermolecular distance. However, a more-direct evidence for H bond-mediated charge transport is afforded by a closer examination at the HOMO versus LUMO transfer integrals in terms of the respective shape of these orbitals. From Fig. 3e, a major difference between the bonding–antibonding pattern of the frontier molecular orbitals is that, while there is a large contribution to the wavefunction originating from NH in the HOMO, this contribution vanishes for the LUMO. In polymorph II, the absence/presence of a node on the NH unit correlates with *J*_HOMO_ being ~4 times larger than *J*_LUMO_ for the H-bonding pair (16.9 vs. 4.4 meV; Supplementary Fig. 4). This clearly points to the positive effect on the transfer integral of spreading the electronic density on the hydrogen bonding NH unit. We further note that for polymorph I, where the close-to-90° dihedral angle electronically decouples the H-bonding pair, smaller and comparable transfer integrals are predicted for the HOMO and LUMO orbitals (7.1 vs. 9.8 meV; Supplementary Fig. 4). These analyses lead us to the conclusion that a low dihedral angle and a close distance of the H-bonding pair together with wavefunction delocalization over the H-bonding moieties act in concert to raise the electronic coupling between molecules along the H-bond direction.

### Fabrication and structure characterizations of highly aligned ellipticine films

To validate theoretical predictions, it is necessary to fabricate highly crystalline, highly aligned ellipticine thin films with controlled polymorphism for measuring intrinsic and extrinsic charge transport properties in field-effect-transistor devices. High degree of alignment and crystallinity will allow us to compare charge carrier mobilities along the π−π vs. the H-bonding directions. The ability to control polymorphs offers the opportunity to determine how varying π−π and H-bonding interactions influence intrinsic charge transport properties. Towards this end, we developed solution-processing methods to controllably access polymorph I and II of ellipticine and prepared highly crystalline samples with high degree of alignment for polymorph II.

**Fig. 4:**
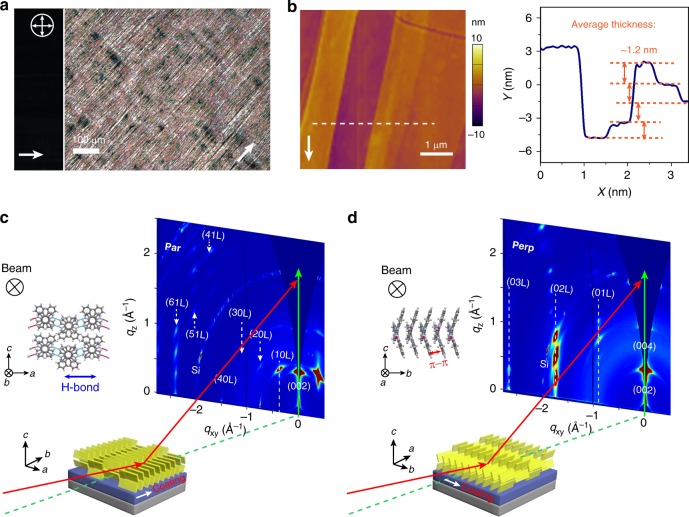
Structural characterizations of ellipticine thin films. The films were prepared via meniscus-guided coating from its 3 mg ml^−1^ THF solution at 0.05 mm s^−1^. **a** Cross-polarized optical microscopy (CPOM) images of ellipticine thin films as deposited (see Methods). **b** AFM height image and the cross-sectional height profile of the printed thin film along the white dotted line. The step heights are ~ 1.1–1.2 nm, comparable to the thickness of a single molecular layer of ellipticine (1.04 nm). The white arrows in panel **a** and **b** denote the coating direction. **c**, **d** Schematic of GIXD experiments and the GIXD patterns. The incident X-ray beam was set **c** parallel and **d** perpendicular to the coating direction. The (hkl) miller indices were labeled for corresponding diffraction peaks or Bragg rods. The inset schematic illustrates the inferred orientation of crystalline domains respective to the incident X-ray beam and the coating direction

We applied the meniscus-guided solution coating method to entrap the neat phase of polymorph II (metastable at room temperature as determined from solvent vapor annealing) by varying coating speeds when deposited from tetrahydrofuran (THF) solution on plasma-treated Si wafer (Supplementary Fig. 9). Using this method, the kinetic stability of metastable forms can be substantially enhanced by the combined mechanism of nanoconfinement and kinetic trapping as demonstrated in our previous works^30,34–37^. The alignment, crystallinity, and polymorph identity of the solution coated ellipticine thin films were characterized using a combination of cross-polarized optical microscopy (CPOM), atomic force microscopy (AFM) and grazing incidence X-ray diffraction (GIXD) (Fig. 4, Supplementary Figs 10–12). Using CPOM, we observed millimeter to centimeter sized crystalline domains that extinguished cross-polarized light at the same time, indicative of high degree of alignment (Fig. 3a). AFM revealed well-defined crystalline ribbons and a terraced topology, which are characteristics of high crystallinity (Fig. 4b and Supplementary Fig. 12b). A single terrace height is 1.1–1.2 nm, comparable to the thickness of one molecular layer (1.04 nm) along the *c* axis of polymorph II. This indicates that the *c* axis is oriented normal to the substrate plane, confirmed by GIXD. We observed sharp, well-defined diffraction peaks from GIXD patterns. GIXD peak indexing against the single-crystal structures led to assignment of polymorph II to the solution coated ellipticine thin films (Supplementary Fig. 11). By comparing the diffraction patterns probed parallel (par) and perpendicular (perp) to the coating direction, the orientation of the crystalline domains was further analyzed as the following (Fig. 4c, d). First, for both sample orientations (par and perp), the GIXD patterns showed out-of-plane diffraction peaks at *q*_z_ = 0.61 Å^−1^, corresponding to the (002) plane spacing of polymorph II. This indicates that the *c* axis is normal to the substrate plane, consistent with the AFM inference. With regard to in-plane diffraction features, (10 L), (20 L), and (30 L) Bragg rods appeared only in the perpendicular scan; at the same time, (01 L), (02 L), (03 L), and (04 L) Bragg rods were observed only in the parallel scan. This phenomenon points to the high degree of in-plane alignment in crystalline thin films prepared via meniscus-guided coating, consistent with the CPOM observation. It can be further deduced that the *b* axis (π−π stacking) is oriented parallel to the coating direction and the *a* axis (H bonding) perpendicular to coating (Fig. 4c, d). In addition to coating from THF solutions, aligned crystalline thin films of polymorph II were also obtained when coating from dimethyl sulfoxide (DMSO) solutions (Supplementary Figs 10b and 12).

Neat crystals of polymorph I were also fabricated, but using slow evaporation from bulk DMSO solution instead (see Methods). Owing to its slow nucleation and growth kinetics, neat polymorph I was not accessible using meniscus-guided coating; instead, mixed phases of I and II were obtained even at low coating speeds. Aligned crystalline films of polymorph I was prepared by laminating needle-shaped single crystals of polymorph I onto dielectric substrates for charge transport property characterizations (Supplementary Fig. 13a). Single-crystal diffraction confirmed that the long axis of the crystal is parallel to the *a*-axis which is along the π−π stacking direction (Supplementary Fig. 13b).

### Measuring intrinsic charge carrier mobility

**Fig. 5:**
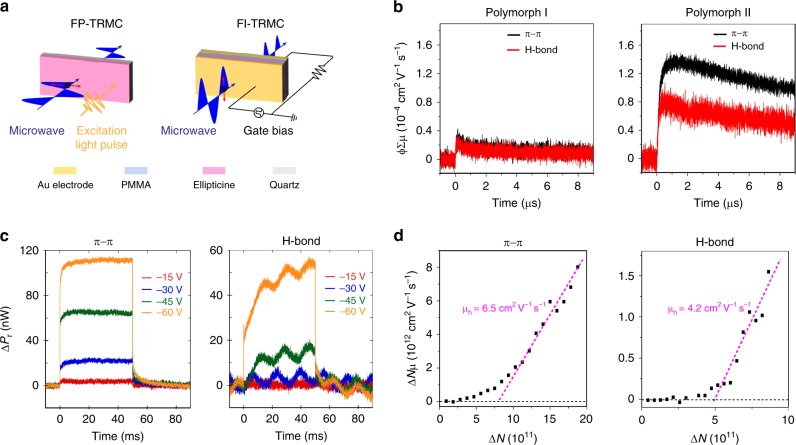
Intrinsic carrier transport properties via microwave conductivity. **a** Schematic of flash-photolysis and field-induced time-resolved microwave conductivity (FP- and FI-TRMC) techniques. **b** FP-TRMC response of ellipticine samples of polymorph I and II. ϕ∑μ denotes the product of charge separation quantum yield (*ϕ*) and the sum of photo-generated hole and electron mobilities (∑μ); ϕ∑μ is proportional to photoinduced conductivity change. **c** Gate bias effect on FI-TRMC signals for hole transport along the π−π stacking and the H-bonding directions. Δ*P*_r_ denotes the change in the power of reflected microwave during gate bias pulsing; Δ*P*_r_ is proportional to field-induced conductivity change. **d** Correlation between holes number Δ*N* and pseudo electrical conductivity Δ*Nμ* along the π−π stacking and H-bonding directions

Aligned ellipticine samples were used for measuring intrinsic charge carrier mobility using two noncontact microwave-based techniques: flash-photolysis and field-induced time-resolved microwave conductivity, abbreviated as flash-photolysis time-resolved microwave conductivity (FP-TRMC) and field-induced time-resolved microwave conductivity (FI-TRMC), respectively. TRMC measures short-range, nano-scale mobility of charge carriers resonant with an oscillating microwave electric field; the measured mobility is free from grain boundary and contact effects^38,39^. In the FP-TRMC technique (Fig. 5a), charge carriers are generated by a photoinduced charge separation process, and resonate as they absorb the incident microwaves, resulting in a change in the power of the reflected microwaves Δ*P*_r_ whose kinetic traces are monitored. Owing to photoexcitation induced ionization, both holes and electrons are generated together. Complementing to FP-TRMC, FI-TRMC can evaluate hole and electron mobilities separately at the semiconductor/dielectric interface using the metal insulator-semiconductor (MIS) structure (Fig. 5a). In this technique, the electrons or holes are induced by applying a positive or negative gate bias (hence, the name field-induced) rather than through photolysis.

Using the FP-TRMC technique, we compared charge carrier mobilities of the two polymorphs, as the technique is applicable to both single-crystal and thin film samples. Monitoring the kinetic traces of Δ*P*_r_ informs the transient photoconductivity of the generated charge carriers Δ*σ*, which is proportional to Δ*P*_r_/*P*_r_. Owing to the electric field polarization of the microwaves^40^, the in-plane anisotropy of the photoconductivity in aligned samples can be measured. For data analysis, Δ*σ* is evaluated as *ϕΣμ* where *ϕ* is the quantum yield of charge carrier generation per absorbed photon and Σμ is the sum of hole and electron mobilities^41^. Shown in Fig. 5b, the (*ϕΣμ*)_max_ value of polymorph II is ~ 7 times that of polymorph I along the π−π stacking direction, and ~4 times that of polymorph I along the H-bonding direction. In comparison, the calculated II/I mobility ratios are ~2.5 and 3 along the π−π stacking and the H-bonding direction, respectively. For polymorph II, the total charge carrier mobility measured through the H-bond is significant considering the absence of π orbital overlap, albeit lower than through the π−stack given the same *ϕ*. For comparison, we applied the same technique to thermally evaporated pentacene thin film – a benchmark organic semiconductor, and obtained similar (*ϕΣμ*)_max_ values as the polymorph I case (Supplementary Fig. 14). We note that owing to the low density of photoinduced charge carriers in these materials, precise evaluation of charge carrier mobility using FP-TRMC is challenging^42,43^.

We next evaluated intrinsic hole mobilities using FI-TRMC. For sample fabrication in the MIS geometry, polymorph II thin films were solution coated on poly(methyl methacrylate) (PMMA) dielectric substrates, which showed exceptionally high degree of alignment (Supplementary Fig. 15). Limited by the MIS geometry, we were not able to use polymorph I single-crystal needles in this technique. Figure 5c presents the gate bias-dependent Δ*P*_r_ kinetic traces along the π−π stacking direction vs. the H-bonding direction. Δ*P*_r_ is proportional to the change in the pseudo electrical conductivity Δ*Nμ*, wherein Δ*N* is the change in charge carrier number^43^. In the MIS geometry, Δ*N* of injected carriers is calculated from the applied voltage Δ*V* using Δ*N* = *C*_ox_Δ*V*/*e*, where *C*_ox_ is the capacitance of the insulator. Hence, the charge carrier mobility *μ* can be extracted from the Δ*N*–Δ*Nμ* plot shown in Fig. 5d. We measured intrinsic hole mobilities as high as 6.5 cm^2^ V^−1^ s^−1^ and 4.2 cm^2^ V^−1^ s^−1^ along the π−π stacking and the H-bonding directions, respectively, which is in reasonable agreement with the theoretical predications (Fig. 2d). Using the same technique, comparable hole mobilities were obtained for pentacene (6.3 cm^2^ V^−1^ s^−1^)^43^ and BBTBDT (4.5 cm^2^ V^−1^ s^−1^)^44^, attesting to the potential of ellipticine serving as a high-performance organic semiconductor and the viability of utilizing H bond pathway for efficient carrier transport. We further deduced interfacial trap density from the *x* axis intercept of the linear region^45^ in Fig. 5d, and obtained 4.2 × 10^12^ cm^−2^ and 2.7 × 10^12^ cm^−2^ measured along the π−π stacking and the H-bonding directions, respectively. These values are on the same order of magnitude but slightly higher than literature reports (~1 × 10^12^ cm^−2^ ^45,46^^,^).

### Application of ellipticine in field-effect transistors and chemical sensors

**Fig. 6:**
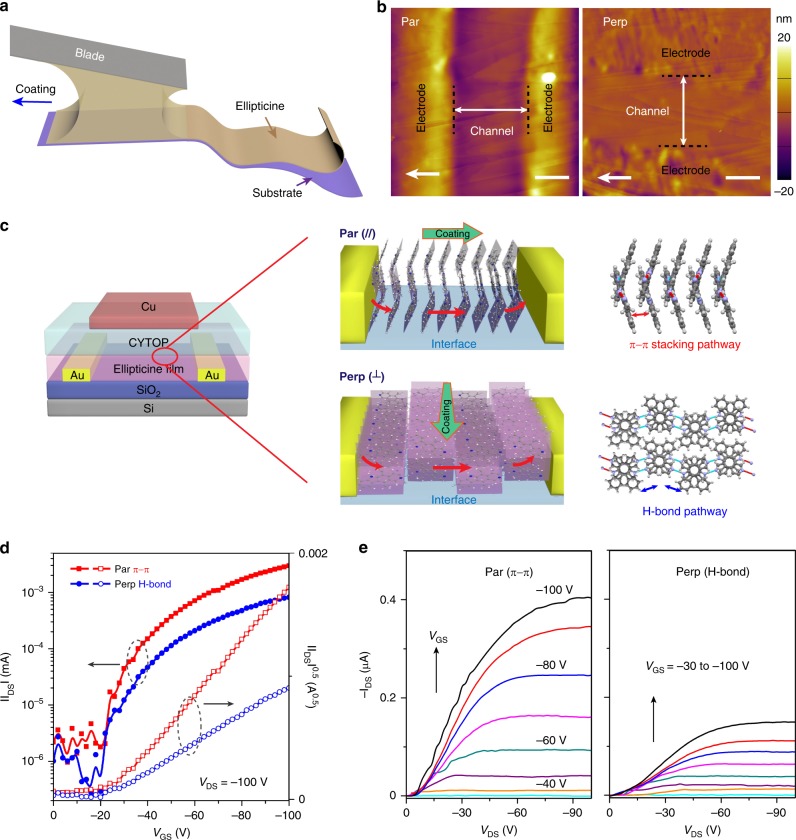
OFET device performance of solution coated ellipticine polymorph II thin films. **a** Schematic of device fabrication via the meniscus-guided solution-coating process. **b** Atomic Force Microscopy (AFM) tapping mode height images of the device channel region with the channel parallel (π−π) and perpendicular (H-bond) to the coating direction. Scale bars are 2 μm. The white arrows denote the coating direction. **c** Schematic of Organic Field-Effect Transistor (OFET) device configuration and molecular orientation in the conductive channels when measured in the parallel or perpendicular direction. The OFET device shown was used in the top-gate bottom-contact (TGBC) geometry with CYTOP as the dielectric layer or in the bottom-gate bottom-contact (BGBC) geometry with SiO_2_ as the dielectric layer. **d**, **e** Transfer and output curves of ellipticine-based TGBC OFET. Performance of BGBC devices are shown in Supplementary Fig. 17

To evaluate long-range charge transport over device-relevant length scales and to demonstrate potential for practical applications, we fabricated OFETs with the conductive channel along the π−π stacking and the H-bonding directions of ellipticine polymorph II (Fig. 6). The ellipticine layer was deposited via meniscus-guided solution coating on SiO_2_ substrates pre-patterned with Au source-drain electrodes of 5–10 μm channel lengths. The as-deposited crystalline films exhibited domain sizes spanning hundreds of microns to millimeters in width, sufficiently large to cover an entire OFET device (Supplementary Fig. 16). The high degree of alignment was evident from the observation that the part of the film covering the device channel region extinguished cross-polarized light at once, consistent with the GIXD results (Fig. 3c, d, and Supplementary Fig. 12c). AFM further revealed a single-crystalline domain bridging the source-drain electrode (Fig. 6b). These morphology characteristics allow us to compare device performance along the π−π and H-bond directions directly (Fig. 6c).

The OFET device performance in various device configurations was summarized in Fig. 6, Supplementary Table 3, 4 and Supplementary Fig. 17. Fig. 6d, e show the representative transfer and output curves of top-gate bottom-contact devices fabricated via solution process, which yielded apparent hole mobilities of up to 1.3 ×10^–3^ cm^2^ V^−1^ s^−1^ and 0.4 × 10^–3^ cm^2^ V^−1^ s^−1^ along the π−π stacking and H-bonding directions, respectively. Here, we emphasize that the OFET device performance is strongly affected by the enormous charge injection barrier at the semiconductor/electrode interface owing to the deep HOMO level of ellipticine; the resulting large contact resistance is manifested in the S-shaped output curves at low *V*_DS_. On top of that, there exists a high interfacial trap state density exceeding 1.5 × 10^13^ eV^−1^ cm^−2^ for top-gate devices estimated from the subthreshold swing region of the transfer curve (see Methods), which is substantially higher than the values reported in high-performance small molecule organic semiconductor OFET devices (~ 10^12^ eV^−1^ cm^−2^)^47,48^. Such high-trap density may be induced by trace amount of water or residual polar solvents^49,50^. Both factors combined, the apparent mobility is three orders of magnitude lower than the intrinsic mobility measured by TRMC techniques. Exhaustive optimization of FET device performance is not the focus of this work and is therefore beyond the scope. Nonetheless, the measured charge transport anisotropy along the π−π stacking vs. the H-bonding directions is consistent with the theoretical prediction and TRMC results. This is one of the few reports on measurable hole transport along the H-bonding direction over microns length scale in dry solid-state electronic devices. We note that proton transport through H-bond networks have been demonstrated in field-effect transistors^51^, however, we ruled out the mechanism of proton transport in our case (Supplementary Fig. 18).

We further demonstrated ellipticine-OFET-based flexible sensor chip for ethyl acetate gas sensing (Supplementary Fig. 19). Breath ethyl acetate is a biomarker for lung cancer^52^, but it remains challenging to detect ethyl acetate at low concentration owing to its low reactivity with most organic semiconductors^53^. Typical OFET-based sensors for ethyl acetate vapor usually exhibit very low current response of up to 3% at 200 ppm^54,55^. In comparison, the ellipticine devices show a high sensitivity with Δ*I*/*I*_0_ over 8% even at concentration as low as 1 ppb. The sensitive detection of ethyl acetate vapor is attributed to possible formation of H-bond between the amine moiety of ellipticine and the carbonyl group of ethyl acetate.

## Discussion

In this work, we discovered promising H-bonded organic semiconductors from DNA topoisomerase inhibitors, some of which have been extensively investigated for their anticancer properties but failed the clinical trials. Density functional theory (DFT) calculations and UV–Vis spectroscopy showed that ellipticine, SN38, 10-hydroxy-camptothecin, doxorubicin, ethonafide, and amonafide exhibit electronic structures that resemble wide-bandgap organic semiconductors, with measured bandgaps of 2.5–3 eV in solution and in crystalline films, indicating their potentially high-environmental stability and optical transparency. Ellipticine was further evaluated due to better energy level alignment between its HOMO and electrode work function. Ellipticine exhibited two crystal polymorphs, both featuring π-stacked columns linked by H-bonds orthogonal to the π−π stacking direction. Notably, the calculated hole transfer integrals and predicted hole mobility reached 16.9 meV and 1 cm^2^ V^−1^ s^−1^ along the H-bonding direction in Polymorph II, and 83.0 meV and 3.7 cm^2^ V^−1^ s^−1^ along the π−π stacking direction. Comparing polymorph I and II indicated that the charge transfer integral along the H-bonding direction is sensitive to the dihedral angle between the molecular planes of the H-bonded pair, which has not been found before. The predicted high mobility in polymorph II was validated by intrinsic charge transport property measurements using time-resolved microwave conductivity, which yielded hole mobility as high as 6.5 cm^2^ V^−1^ s^−1^ along π–π stacking and 4.2 cm^2^ V^−1^ s^−1^ along the H-bonding direction. Separation of charge transport along H-bonding from π–π stacking was enabled by highly aligned, highly crystalline, phase-pure polymorph II thin films prepared by meniscus-guided coating. We further applied printed ellipticine thin films in field-effect transistors and chemical sensors, and demonstrated experimentally long-range hole transport along H-bonds over micron length scales in dry solid-state electronic devices. Owing to high-trap density and charge injection issues, ellipticine-based OFETs yielded hole mobilities of only 1.3 × 10^–3^ cm^2^ V^−1^ s^−1^ and 0.4 × 10^–3^ cm^2^ V^−1^ s^−1^ along the π−π stacking and H-bonding directions. Nonetheless, we show the exciting potential to discover organic electronics with unique materials chemistry by re-purposing failed drug candidates.

## Methods

### Materials

Ellipticine (99% by high-performance liquid chromatography, HPLC) was purchased from Fisher Scientific Co LLC. SN38, 10-hydroxy-camptothecin and amonafide (> 99% by HPLC) were purchased from Selleckchem. Dimethyl sulfoxide (AR, ACS agent) and tetrahydrofuran (AR, ACS agent) were purchased from Thermo Fisher Scientific. PMMA (*M*_w_ = 120 kg mol^−1^), *n*-butylacetate (anhydrous), Octadecyltrichlorosilane was purchased from Sigma-Aldrich. CYTOP and diluted solvent were purchased from Asahi Glass Co., LTD. All the materials and solvents were used as received.

### Sample preparation

For single-crystal X-ray diffraction, single crystals of polymorph I and II were prepared via drop-casting on glass substrates from 10 mg ml^−1^ and 5 mg ml^−1^ DMSO solutions followed by slow evaporation at the room temperature; 10 and 5 mg ml^−1^ solutions yielded polymorph I and II single crystals respectively, both took the form of long needles. For charge transport property measurements, single-crystalline needles of ellipticine polymorph I were grown from 1 ml, 0.05 mg ml^−1^ DMSO solution via slow evaporation over 2 weeks, harvested using micromount with the aperture size of 1 mm after removing > 90% solvent and transferred onto quartz glass substrates with the long axes of the needles aligned in the same direction. Thin films of ellipticine polymorph II were prepared using the meniscus-guided coating method, following a similar protocol as reported in our previous work^47,56^. Specifically, ellipticine/THF solution (3 mg ml^−1^) was used to prepare thin films on O_2_ plasma treated Si or SiO_2_/Si substrates. The substrate temperature was set at 25 °C. The coating speed was 0.05 mm s^−1^. 20 wt% PMMA (with respect to the weight of ellipticine) was added in the solution to effectively eliminate the cracks in crystalline domains and to reduce the trap density at the semiconductor/dielectric interface beneficial for field-effect transistor device measurements. In certain cases, ellipticine/DMSO solution (2 mg ml^−1^, 10 wt% PMMA) was also used to solution coat ellipticine thin film of polymorph II on plasma treated Si and SiO_2_ substrate for film characterizations. The substrate temperature was 100 °C and the coating speed was 0.01 mm s^−1^. We chose THF for sample preparation for experimental charge transport property characterizations, as DMSO dissolves PMMA dielectrics needed for FI-TRMC measurements.

### Computational methods

For electronic structure predictions, DFT calculations were performed on isolated drug molecules in the gas phase as described elsewhere^57^. The geometries were optimized at the B3LYP level with the 6–311 + G(d, p) basis sets using the Gaussian 09 package^58^. We compared semilocal functional B3LYP with long-range corrected hybrid functionals CAM-B3LYP and ωB97^59^. For ellipticine, we obtained HOMO/LUMO levels of −6.78/−0.60 eV and −7.80/−0.36 eV using CAM-B3LYP and ωB97, respectively. The B3LYP gave closer values to experimental results, possibly due to low degree of conjugation for this class of small molecules and limited delocalization error using the semilocal hybrid functionals.

The charge transfer integrals and charge transport properties were calculated in the hopping regime assuming that charges are hopping between neighboring molecules. The reorganization energy *λ* (sum of inner reorganization energy of the molecule (*λ*_i_) and the reorganization energy of the surrounding medium (*λ*_S_)), which reflects the degree of geometric relaxation energy of the charged species, was also evaluated at the DFT level for a single molecule in vacuum based on the procedure described elsewhere^60^. The details on carrier transfer within the hopping model are calculated as below: Firstly, the rate of carrier transfer (*k*_hop_) is determined with the Marcus–Levich–Jortner equation:^60,61^

1 $$k_{hop} = \frac{{4\pi ^2}}{h}J^2\frac{1}{{\sqrt {4\pi \lambda _Sk_BT} }}\mathop {\sum}\nolimits_\upsilon {{\mathrm{exp}}( - S)\frac{{S^\nu }}{{\nu !}}{\mathrm{exp}}} \left[ { - \frac{{(\lambda _S + {\bf{\nu }}\hbar \omega + \Delta G^0)^2}}{{4\lambda _Sk_BT}}} \right]$$

where *J* represents the transfer integral, the external reorganization energy *λ*_*S*_ is set to 0.1 eV and ℏ represents an effective vibrational mode that assists charge transport set at 0.2 eV^62^, **ν** is the net drift velocity of the charge carriers, *k*_*B*_ is the Boltzmann constant, *T* is the temperature, *S* is the Huang–Rhys factor defined as λ_i_/ℏ, ΔG^0^ is the free energy of the reaction. Charge transfer integral, *J* was estimated reliably at the DFT level (PBE/DZ) using the Amsterdam Density Functional package^63^ as described in previous reports^64,65^, describing the amplitude of the interactions between HOMO levels of the neighboring molecules involved in the carriers transfer. ΔG^0^ is expressed as *e***Ḟḋ**, where **Ḟ** and **ḋ** represent the electric field and distance vectors between mass centers, respectively. Thereafter, the hopping time *t*_*ij*_ between neighbor molecules *i* and *j* can be described as *t*_*ij*_ *=* *-ln(r)/k*_*ij*_ while the random number *r* is chosen between 0 and 1. Finally, the mobility was evaluated via the Kinetic Monte Carlo technique with the First Reaction Method algorithm *μ* = *d*_tot_/*t*_tot_*F*, with *d*_*tot*_ total distance traveled and *t*_tot_ total time of during the simulation, *F* is the amplitude of the electric field applied in the simulation as 10,000 V cm^−1^.

To check if the hydrogen bonds contribute to the high value of the transfer integrals, we have first compared the calculated transfer integrals for one dimer extracted from the crystalline phase and for the same dimer but decreasing the NH bond length by 0.1 Å; therefore, weakening the hydrogen bond. The impact of the hydrogen bonds has also been investigated by comparing the calculated transfer integrals of two dimers where one hydrogen bond is present or not, while keeping the relative orientation of the molecules similar. In practice, in the first case, we extracted one dimer from the crystalline structure and replaced the nitrogen atom involved in the hydrogen bond by a CH group. Then, we shifted the molecules apart along the original NH…N direction such that the facing hydrogen atoms of the CH and NH groups are separated by twice the van der Waals radius of one hydrogen atom (*r*_VDWH_ = 1.20 Å). In the second case, the relative orientation of the molecules in the dimer is kept the same but now we replaced the added CH group by one nitrogen atom. The second dimer therefore looks like the dimer extracted from the crystalline structure except that the molecules are more distant from each other. We further calculated hole charge transfer integral J_HOMO_ along hydrogen-bonding direction as a function of NH…N distance, so as to validate the hypothesis that the role of the H-bond is to bring closer the molecular pairs engaged in the H-bond to facilitate overlap of the electronic wavefunction of the conjugated molecular backbone.

### Morphology characterizations

The single-crystal X-ray diffraction for polymorphic crystals were collected with a Bruker D8 Venture equipped with a four-circle kappa diffractometer and Photon 100 detector. The crystal structures were phased by direct methods and refined by full matrix least-squares methods using the software package SHELX-2014-3 (Sheldrick, 2008). The cif files can be obtained free of charge from the Cambridge Crystallographic Data Center as CCDC ELLIPT (polymorph I), and CCDC 1817466 (polymorph II).

The CPOM detections of ellipticine films were using Nikon Ci-POL. Agilent Cary 60 UV-vis spectrometer was used to collect the solution-state and solid-state UV-visible absorptions. The film topology was characterized using an Asylum Research Cypher (Oxford Instruments). GGIXD measurements for thin films were carried out at beamline 8-ID-E (with beam energy of 10.91 keV) at the Argonne National Laboratory. For the measurements, the samples on Si substrate were detected with a 228-mm sample-to-detector distance under helium atmosphere. The diffraction patterns were collected with the film coating direction put parallel and perpendicular to the X-ray. The incidence angle was 0.14° and the exposure time was 5 s. Data analysis was performed using the software GIXSGUI.

### Charge transport property measurements

FP-TRMC measurements were performed with the samples prepared on quartz with a 355 nm harmonic generator (INDI-HG Nd:YAG laser to create a photon density of 4.5 × 10^15^ photons cm^–2^ pulse^–1^). The laser pulses are set with a 5–8 nm pulse duration with a frequency of 10 Hz. The detection was carried out with a probing microwave in a TE_102_ microwave cavity, with the frequency and power of ca. 9.1 GHz and 3 mW, respectively. The signals were amplified by an amplifier system (Micro Denshi CA89-441 FET), and then collected with a Tektronix model TDS3032B digital oscilloscope. Hence, the conductivity transients *ϕΣμ* (*ϕ* is the quantum yield of the photocarrier generation and *Σμ* reflects the sum of the charge carrier mobilities) can be defined as *ϕΣμ* *=* (1/*eAI*_0_*F*_light_)(Δ*P*_r_/*P*_r_), where *e*, *A*, *I*_0_, *F*_light_, Δ*P*_r_, and *P*_r_ represent the elementary charge, the sensitivity factor (S^–1^ cm), the incident photon density of the excitation laser (photon cm^–2^), the filling factor (cm^–1^), the changed and the steady microwave power reflection from the cavity^41^.

FI-TRMC measurements, by contrast, were performed with the X-band microwave apparatus^66^ adjusted with a signal generator (SMF 100 A, Rohde Schwarz). The MIS device (active area of 6.0 × 3.0 mm^2^) was fabricated with Au electrode/Ellipticine/electrode, and loaded gate bias with a multifunction generator (WF1973, NF Corporation) and amplified by a high-voltage amplifier (T-HVA02, Turtle Industry). The MIS device was set in the microwave cavity (located at the center of the 23×10 mm^2^ waveguide), and the voltage induced signals were amplified was amplified by an RF amplifier (CA812-304, Ciao Wireless). A digital phosphor oscilloscope (MDO 3022, Tektronix) was used to detect the microwave signal and the injection current. The pseudo-conductivity (Δ*Nμ*) was evaluated with the equation, Δ*Nμ* = (*S*/*eA*)(Δ*P*_r_/*P*_r_)(d*F*_*gate*_/*d*)^−1^, where *μ*, *N*, *S*, *A*, *F*_*gate*_, and *d* denote the charge carrier mobility, number of charge carriers, area of the charged region, sensitivity factor, filling factor, and thickness of the charged region, respectively^67^.

OFETs were fabricated with bottom-gate bottom-contact and top-gate bottom-contact (TGBC) structures. The ellipticine films were deposited on plasma treated 300 nm SiO_2_/Si substrate. Patterned on the substrate were source-drain electrodes of 25 nm Au with 5 nm Ti adhesion layer; the channel length and width were 5 μm and 1400 μm, respectively. F_4_-TCNQ doping were performed via spin coating the water/acetone (9:1 in volume ratio) solution on a spinning substrate (*r* = 4000 rpm) and stored in vacuum overnight before measurement. The dopant concentration ranged from 0.25 mg ml^−1^ to 2 mg ml^−1^. For TGBC structure, CYTOP (3:1 diluted) was spin-coated on top of the ellipticine layer at 2000 rpm for 1 min and then thermal annealed at 100 °C for 30 min. Thereafter, patterned Cu film (40 nm) was vacuum deposited as gate electrode. A Keysight B1500A instrument was employed for the transistor characterization. The mobility *μ* was extracted by performing linear regression in the saturation regime on *I*_DS_^0.5^ vs. *V*_GS_ plots, following the equation *I*_*DS*_ *=* *(μWC*_*ox*_*/2* *L)(V*_*GS*_*-V*_*T*_*)*^*2*^, where *L* and *W* are the channel length and channel width, respectively. *V*_*T*_ is the threshold voltage. The interfacial trap state density (*N*_it_) was calculated following the equation^48,68^ *N*_*it*_ = *C*_ox_(*S*×e/(*k*_B_*Tl*n10)−1)/*e*^2^, where *S* represents the subthreshold swing, *e* is the elementary charge, *k*_*B*_ is the Boltzmann constant and *T* is the absolute temperature. The subthreshold swing is defined as *dV*_*GS*_/*d(logI*_*DS*_*)*^69^.

Flexible OFET were fabricated on indium tin oxide-coated PET substrate (127 μm). A 350 nm CYTOP layer was spin-coated on the cleaned substrate as dielectric layer (*r* = 2000 rpm and cured at 100 °C for 30 min). In total, 30 nm Au was deposited as source-drain electrodes with shadow mask. Thereafter, ellipticine film were coated on plasma treated CYTOP with 2 mg ml^−1^ DMSO solution at 100 °C. The coating speed was 0.01 mm s^−1^. The chemical sensing was carried out using Keysight B1500A in real time, and the ethyl acetate vapors were injected onto device with syringe. The sensor response was presented as *ΔI/I*_*0*_ *=* *(I’−I*_*0*_*)/I*_*0*_, where *I*_*0*_, *I’*, and *ΔI* are the initial drain current, current after exposure to analyte and the net current change, respectively.

## Supplementary information

Supplementary Information

## Data Availability

All the data support this study are available on request from the author.
