# Supplementary material for: Repurposing DNA-binding agents as H-bonded organic semiconductors
Source: Nat Commun. 2019 Sep 16;10:4217. doi: 10.1038/s41467-019-12248-9 (PMC6746806; doi:10.1038/s41467-019-12248-9)
Supplement: Supplementary file 1 — Supplementary Information [file 41467_2019_12248_MOESM1_ESM.pdf]

Supplementary Information for

Repurposing DNA Binding Agents as H-bonded Organic Semiconductors

Fengjiao Zhang[1,2#], Vincent Lemaur[3#], Wookjin Choi[4,5], Prapti Kafle[1], Shu Seki[4], Jérôme Cornil[3], David Beljonne[3], Ying Diao[1*]

[1]Department of Chemical and Biomolecular Engineering, University of Illinois at Urbana−Champaign, 600 South Mathews Avenue, Urbana, Illinois 61801, United States.

[2]School of Chemical Sciences, University of Chinese Academy of Sciences, Beijing 100049, P.R. China.

[3]Laboratory for Chemistry of Novel Materials, University of Mons, Place du Parc, 20, B-7000 Mons, Belgium

[4]Department of Molecular Engineering, Graduate School of Engineering, Kyoto University, Nishikyo-ku, Kyoto 615-8510, Japan

[5]Department of Chemical Engineering, Center for Advanced Soft Electronics, Pohang University of Science and Technology, Pohang 37673, Korea

[#] These authors contributed equally.

For correspondence

* **E-mail:** yingdiao@illinois.edu

Supplementary Figures

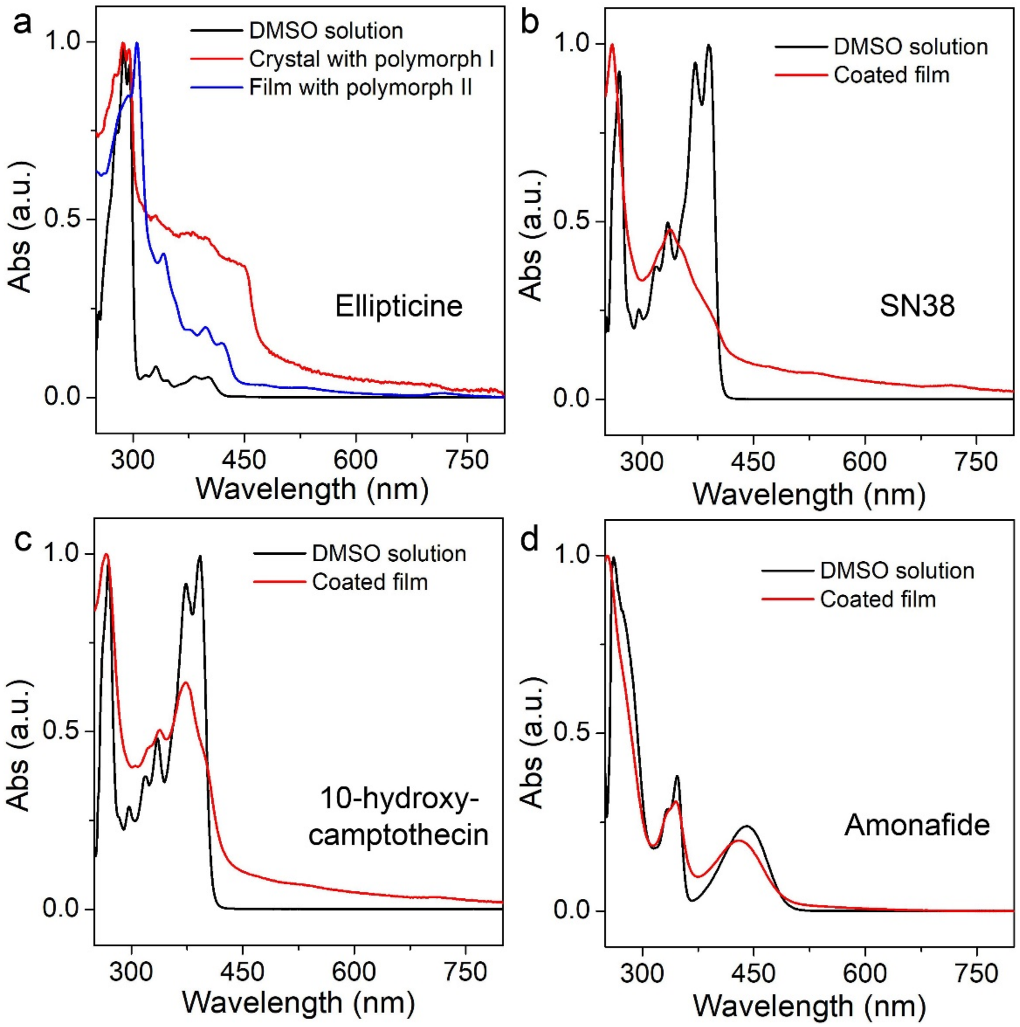

Supplementary Figure 1 | Optical properties of DNA intercalators in dilute solution, single crystal and solution coated thin films. Absorption spectra of (**a**) Ellipticine, (**b**) SN38, (**c**) 10-hydroxy-camptothecin and (**d**) Amonafide. The crystalline films were deposited on plasma treated quartz slides via meniscus-guide coating from DMSO solution (2 mg/ml for ellipticine, and 10 mg/ml for SN38, 10-hydroxy-camptothecin and amonafide). The coating speed was 0.01 mm/s, and the substrate temperature was controlled at 100 °C.

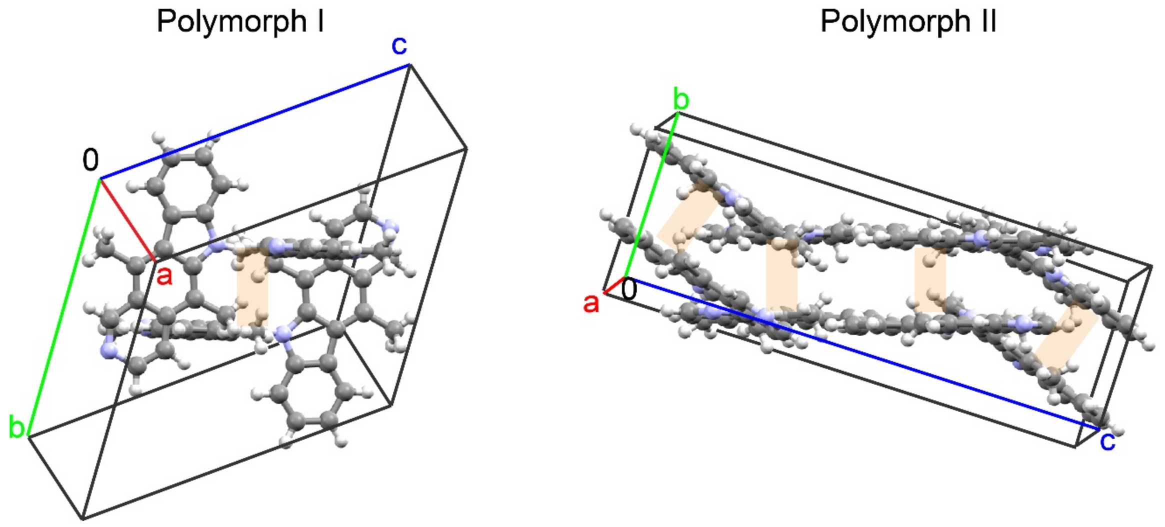

Supplementary Figure 2 | Comparison of the crystal packing for polymorph I and II within one unit cell. The orange shades highlight the overlap between the π planes.

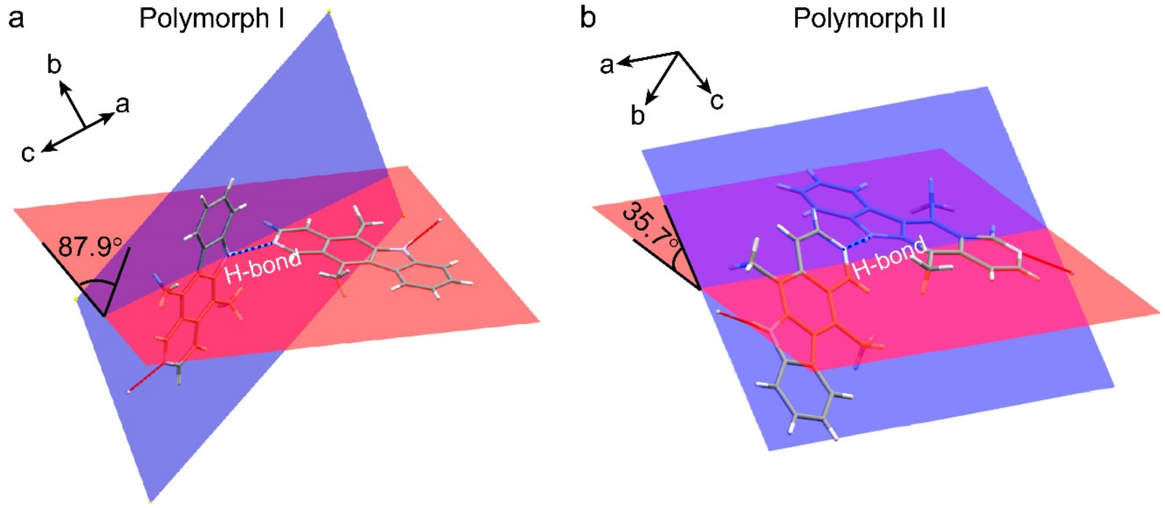

Supplementary Figure 3 | Comparison of dihedral angles. The dihedral angles between the molecular planes of the H-bonding pair shown in (**a**) polymorph I vs. (**b**) polymorph II.

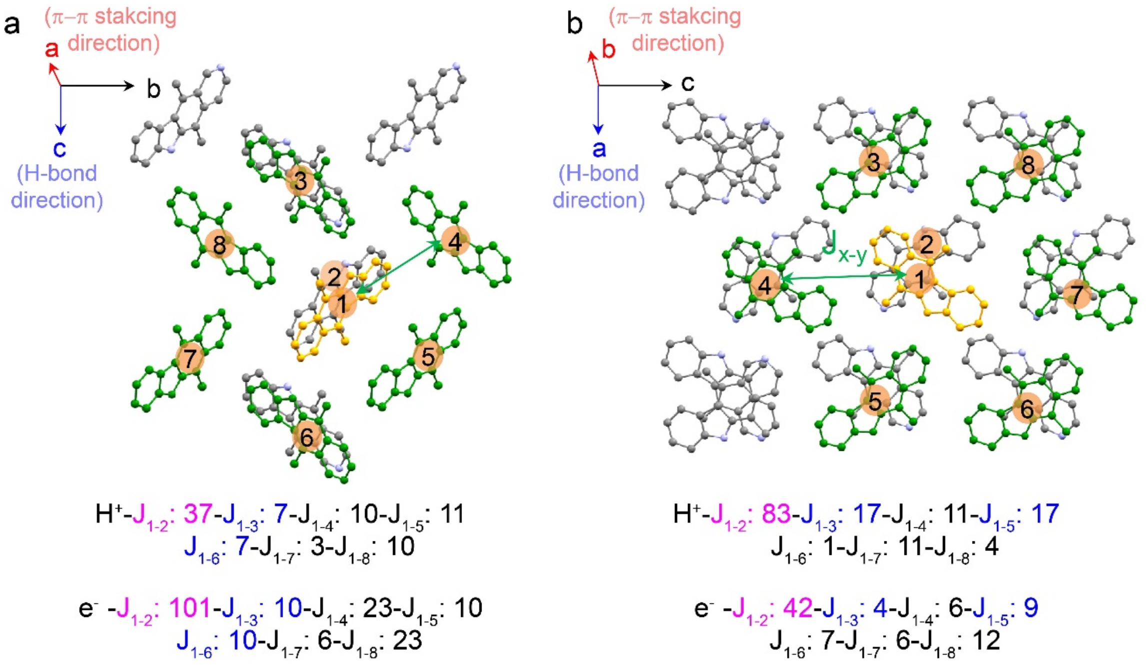

Supplementary Figure 4 | Calculated electronic coupling terms-charge transfer integral J. These graphs summarized the J for hole and electron along different hoping pathways in ellipticine of (**a**) polymorph I and (**b**) polymorph II packing motif. For polymorph I, the molecules along the *a*-axis stack in a co-facial, slip-stacked fashion. Due to the offset between the molecular pairs 1 and 2, J_{12} is calculated as only 36.9 meV for holes but a large value of 100.7 meV for electrons. Along the *c*-axis, there exhibits a large dihedral angle of 87.9° between two adjacent molecular planes (1-3 and 1-6 pairs), leading to a relatively lower J value of <10 meV along the H-bond direction. Along the *b*-axis, there are two herringbone pairs of 1-4 and 1-8 and two co-facial slip-stacked pairs of 1-5, 1-7. All molecular pairs exhibited moderate to low J values due to large intermolecular distances or limited overlap. For polymorph II, the 1-2 molecular pair along *b*-axis exhibits co-facial π–π stacking, corresponding to a large hole transfer integral J_{12} of 83.0 meV. The highest electron transfer integral is also found between the 1-2 molecular pair, which is 41.6 meV. Along the *a* axis, significant J values of 16.9 meV for hole carriers is observed along the H-bonding direction. Compared to polymorph I (J_{13}, J_{16}), the higher J values in polymorph II are attributed to lower dihedral angle of 35.7° between the molecular planes of 1-3 and 1-5, given comparable H-bonding distance in both cases. The charge transfer

integrals for other molecular pairs (1-4, 1-7, 1-8, 1-6) are lower than the $\pi-\pi$ stacking pairs. The transfer integrals for holes and electrons are different due to different degrees of bonding versus antibonding interactions in the overlapping region[1]. An interesting result is that polymorph I exhibits a much higher electron than corresponding hole transfer integral, while polymorph II is the reverse, even though the high electron reorganization energy (λ) characterization introduces higher degree of geometric relaxation for electrons transfer in ellipticine. This may be caused by the molecular stacking change resulting in an electronic structure difference.

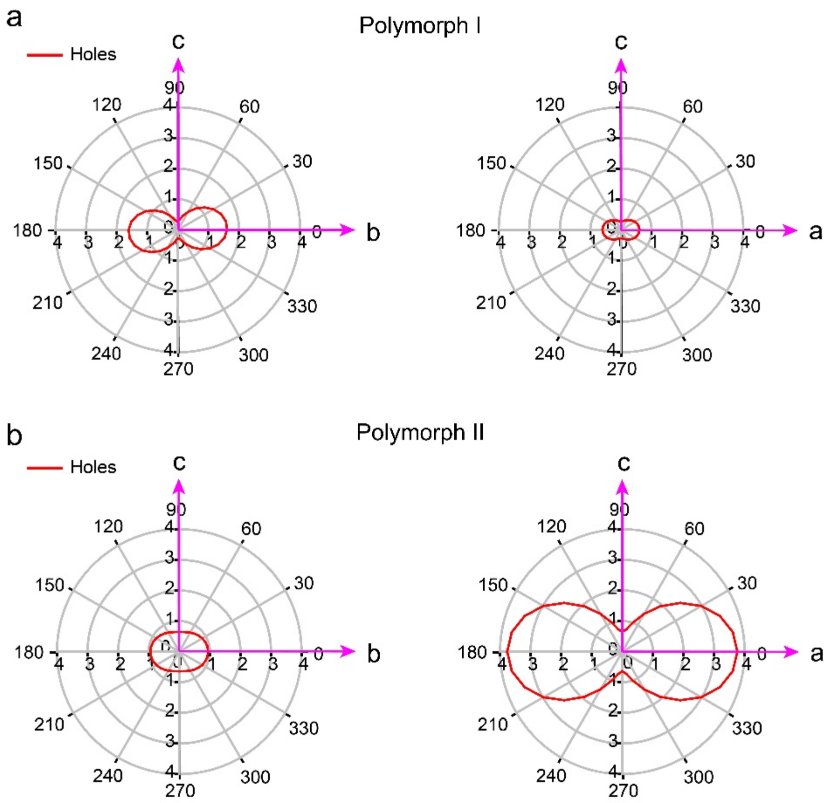

Supplementary Figure 5│ Calculated angle-resolved hole mobilities displayed in the bc and ac planes of ellipticine polymorphs. The unit of hole mobility is $cm^2V^{-1}s^{-1}$ for ellipticine of (**a**) polymorph I and (**b**) polymorph II, as calculated in the hopping regime.

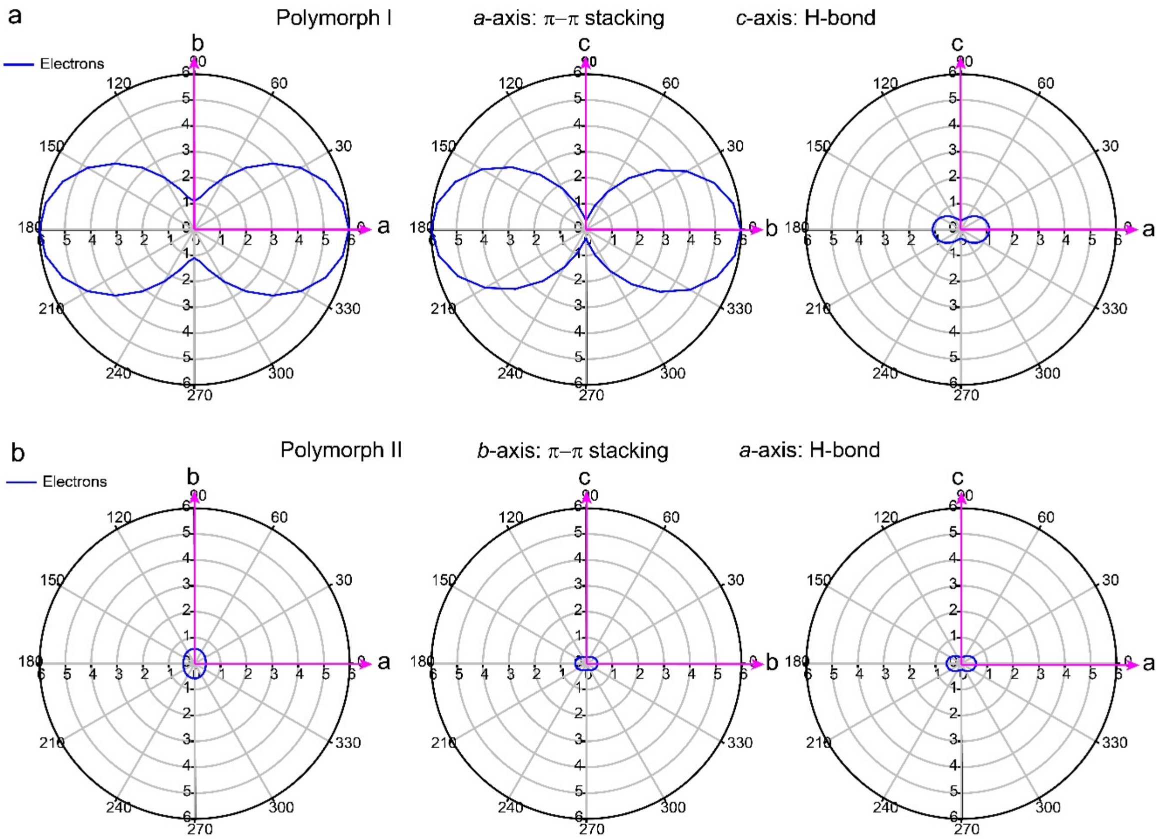

Supplementary Figure 6│ Calculated angle-resolved electron mobilities displayed in ellipticine polymorphs. The unit of electron mobility is $cm^2V^{-1}s^{-1}$ for ellipticine of (**a**) polymorph I and (**b**) polymorph II, as calculated in the hopping regime. The large transfer integral calculated for polymorph I contribute to a high mobility up to 6 $cm^2V^{-1}s^{-1}$ along the π–π stacking direction.

Polymorphs	Original crystal structure	N-H variation
Polymorph I	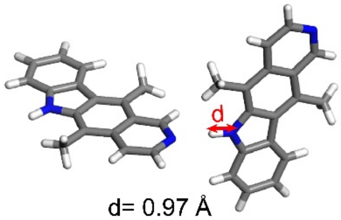<br>d= 0.97 Å<br>J_{HOMO}= 7.1 meV	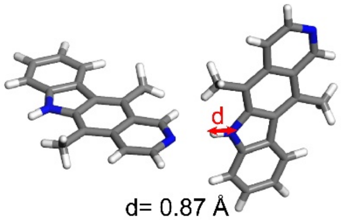<br>d= 0.87 Å<br>J_{HOMO}= 6.8 meV
Polymorph II	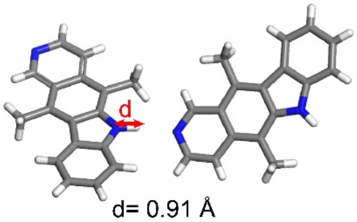<br>d= 0.91 Å<br>J_{HOMO}= 16.9 meV	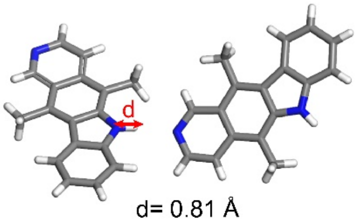<br>d= 0.81 Å<br>J_{HOMO}= 16.5 meV

Supplementary Figure 7 | Effect of the strength of the H-bond on the HOMO charge transfer integral along H-bond direction of ellipticine. The strength of the H-bond is weakened by shortening the N-H bond length by 0.1 Å while keeping the N to N distance (intermolecular distance) fixed.

Polymorphs	Polymorphs	J_{HOMO} along π–π stacking
Polymorph I 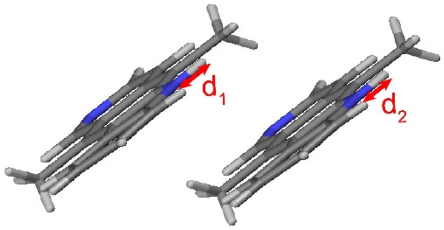	d_1= d_2= 0.97 Å	36.9 meV
	d_1= 0.87 Å, d_2= 0.97 Å	37.0 meV
	d_1= d_2= 0.87 Å	36.4 meV
Polymorph II 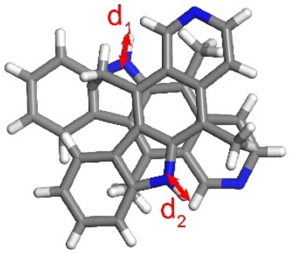	d_1= d_2= 0.91 Å	83.0 meV
	d_1= 0.81 Å, d_2= 0.91 Å	82.1 meV
	d_1= d_2= 0.81 Å	82.7 meV

Supplementary Figure 8 | Effect of the strength of the H-bond on the charge transfer integral of ellipticine along the π–π stacking direction.

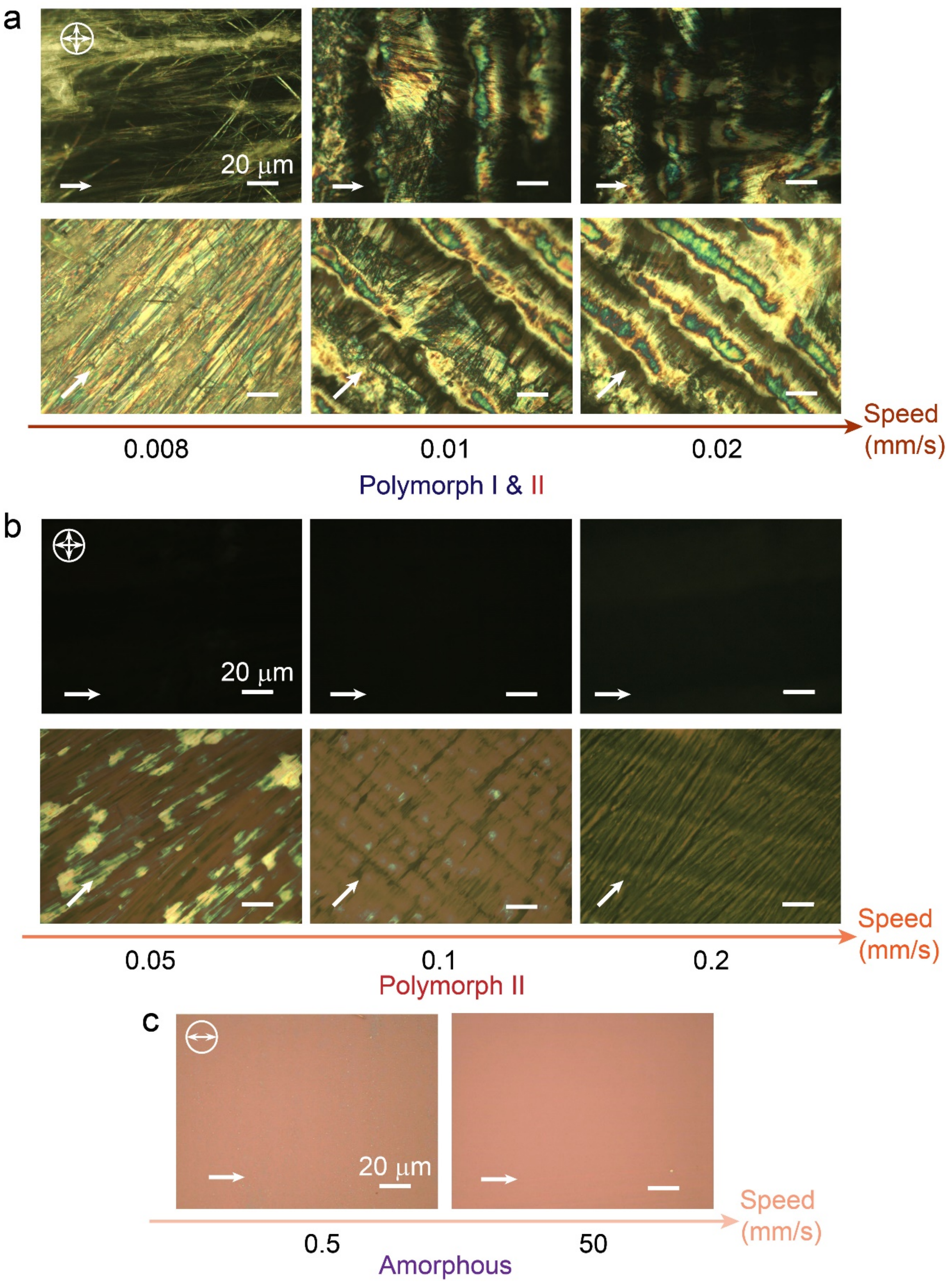

Supplementary Figure 9│ Dependence of polymorphic outcome on coating speed in solution coated ellipticine films. Cross-polarized microscopy images of ellipticine films

at various coating speeds. The arrow indicates the coating direction. All the scale bars are 20 μm. (**a**) At low coating speeds, we observed formation of plate-like polymorph II thin films followed by partial transformation to polymorph I yellow needles atop the polymorph II thin film. Increasing the coating speed from 0.008 mm/s to 0.02 m/s gradually impedes the transition to polymorph I yellow needles. The coated films show low degrees of alignment in this range of coating speed. The polymorph assignment is consistent with our previous study[2]. (**b**) At higher coating speeds (0.05 mm/s to 0.2 mm/s), only plates of polymorph II were observed, oriented along the coating direction indicated by the high birefringence. (**c**) The films become amorphous when the coating speed increased to beyond 0.5 mm/s. The films were coated on plasma treated substrates from THF solution (3 mg/ml with 20 wt% PMMA; the weight percentage of PMMA is with respects to ellipticine). The GIXD diffraction for the films under various polymorphs were previously studied[2], which reflects the controllable molecular stacking via the simple solution coating process.

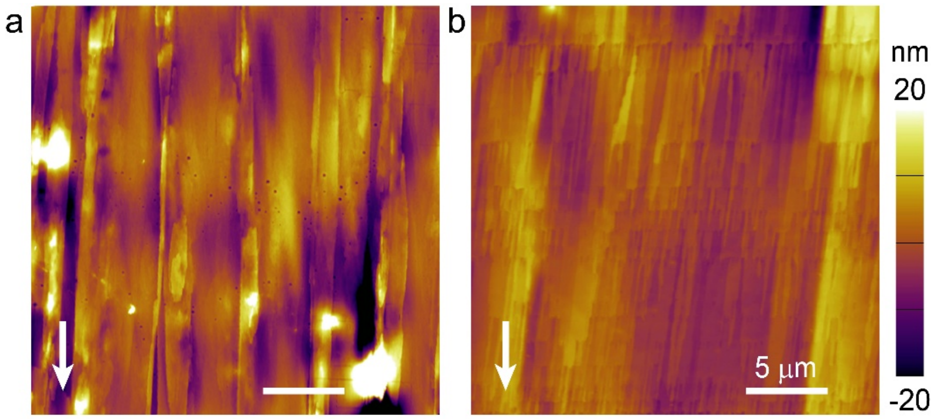

Supplementary Figure 10│ Tapping mode AFM height images of ellipticine thin films. The films were solution coated on plasma treated SiO_2 substrate from (**a**) THF solution with 20 wt% PMMA and (**b**) DMSO solution with 10 wt% PMMA. The PMMA additions ratio is respect to the weight of ellipticine. Large domain sizes were obtained in both cases. For films deposited from THF, the domain width was ~5 μm perpendicular to the blade coating direction. For ellipticine film deposited from DMSO, the domain width was as large as few tens of micrometers. Scale bar: 5 μm. The arrows indicate the coating direction.

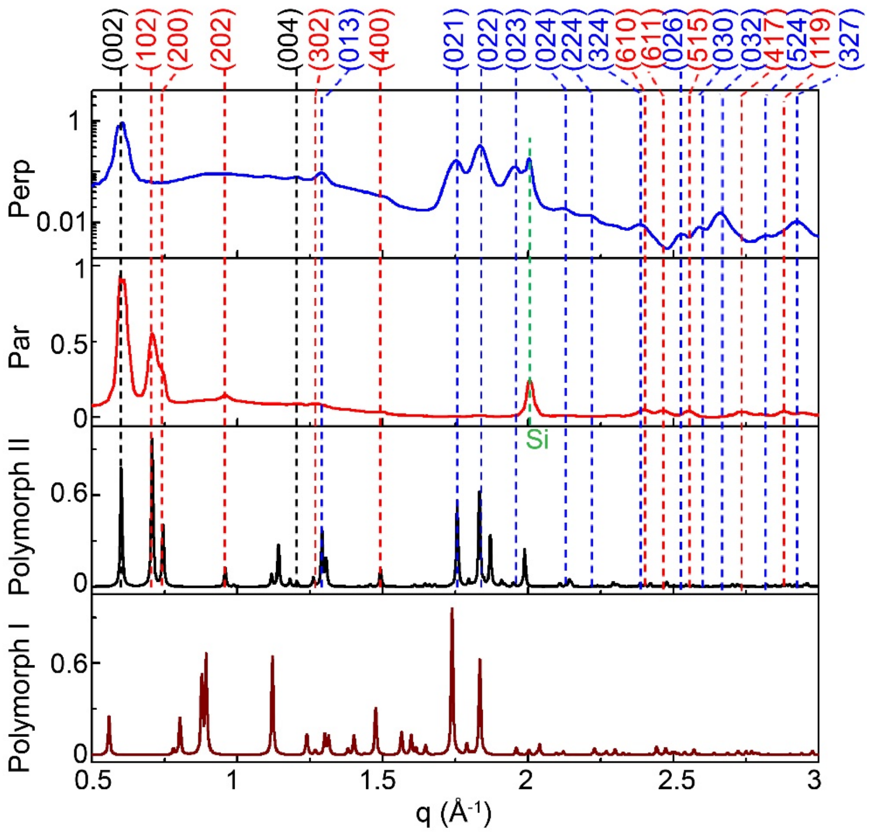

Supplementary Figure 11 | Comparison of GIXD patterns of ellipticine thin film with powder diffraction patterns of ellipticine simulated from single crystal structures. The 1D GIXD patterns correspond to the 2D patterns shown in fig. 3C, D, obtained with the incident beam parallel (Par) and perpendicular (Perp) to the coating direction. The powder XRD patterns were simulated using the Mercury software. The GIXD diffraction patterns were assigned to polymorph II, with the (hkl) miller indices labeled on respective peaks. The exclusive appearance of (h0l) peaks in the parallel scan and of (0kl) peaks in the perpendicular scan is a signature of highly aligned crystalline domains, with the *b*-axis (π–π stacking) parallel to the coating direction and the *a*-axis (H-bonding) perpendicular to the coating direction. The appearance of (00l) peaks in both directions indicate that the *c*-axis is normal to the substrate plane, consistent with the AFM predication.

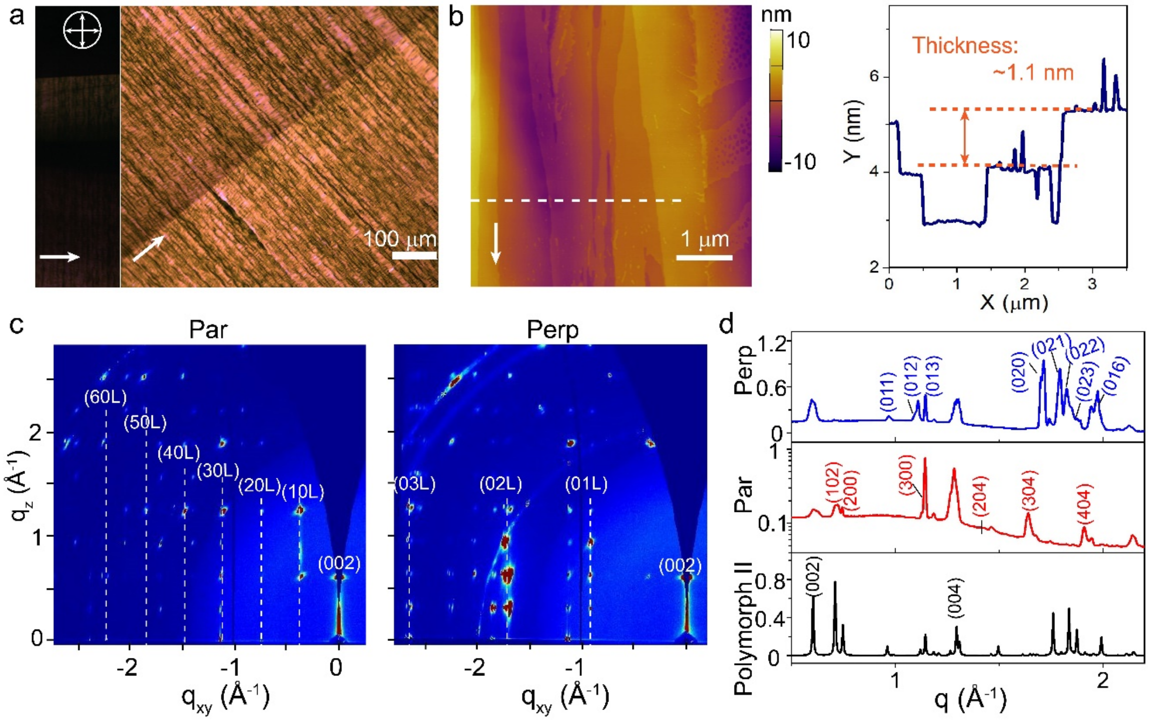

Supplementary Figure 12| Structural characterizations of ellipticine thin films solution coated from DMSO solution. (**a**) C-POM images for ellipticine film deposited from 2 mg/ml DMSO solution at a coating speed of 0.01 mm/s and substrate temperature of 100 °C. (**b**) Tapping mode AFM height image of the coated thin film. The film thickness is 46±5 nm. The cross-sectional height profile along the white dotted line is shown on the left. The white arrows in a and b denote the coating direction. (**c**) GIXD images of the ellipticine thin film with the incidence X-ray beam parallel and perpendicular to the coating direction. (**d**) Comparison of 1D GIXD patterns with the simulated diffraction pattern of ellipticine from polymorph II .cif file. The ellipticine film coated from DMSO was identified as polymorph II.

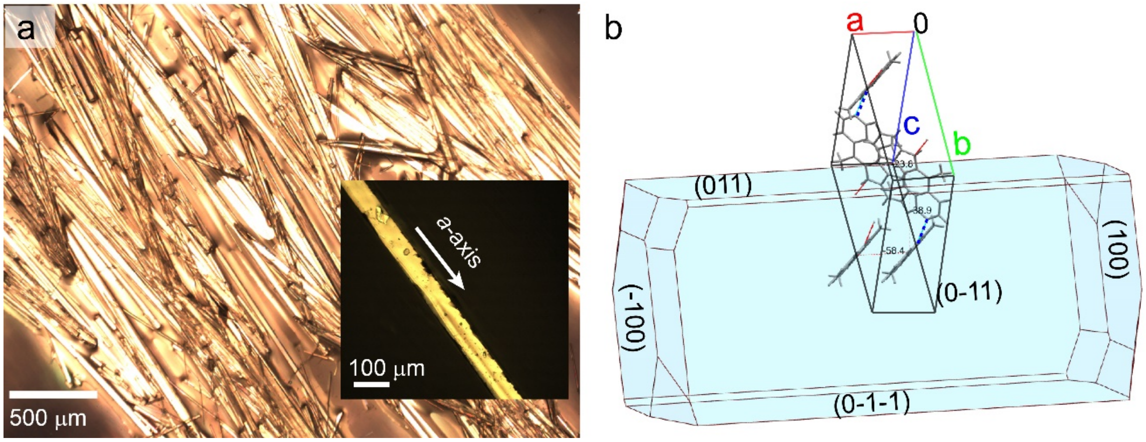

Supplementary Figure 13| Crystalline films of ellipticine polymorph I and crystallographic orientation. (**a**) Optical microscopy image of transferred ellipticine polymorph I films laminated from single crystalline needles grown from bulk DMSO solution. Inset shows the cross-polarized optical microscopy image of a single crystal yellow needle. On average, these needles have a cross section of 50×50 μm^2, and a length reaching several millimeters. (**b**) BFDH morphology of polymorph I single crystals calculated from single crystal structure using the software Mercury. The crystallographic structure of polymorph I was published with crystal structure (CCDC ELLIPT)[3]. The crystal needles exhibit fast growth along the *a*-axis direction. More importantly, the GIXD diffraction of these crystalline needles reflect a comparable diffraction peak with the simulated results, which was reported in previous study[2].

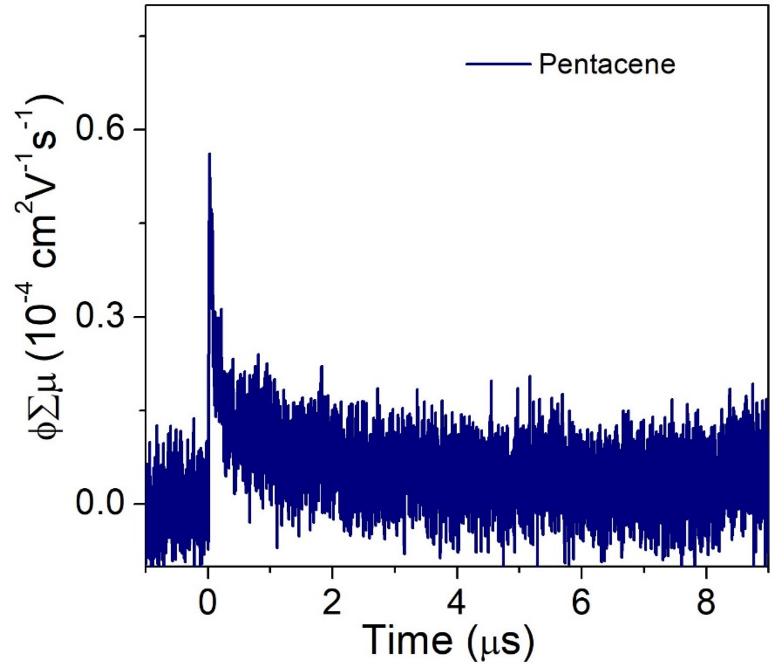

Supplementary Figure 14│ FP-TRMC response of 400 nm pentacene thin film measured in air. The estimated relaxation time τ is ~ 1.5 μs. For comparison, the estimated τ of the photoconductivity of polymorph I and II are ~2 μs and >10 μs, respectively, suggesting a slower charge recombination in the ellipticine samples.

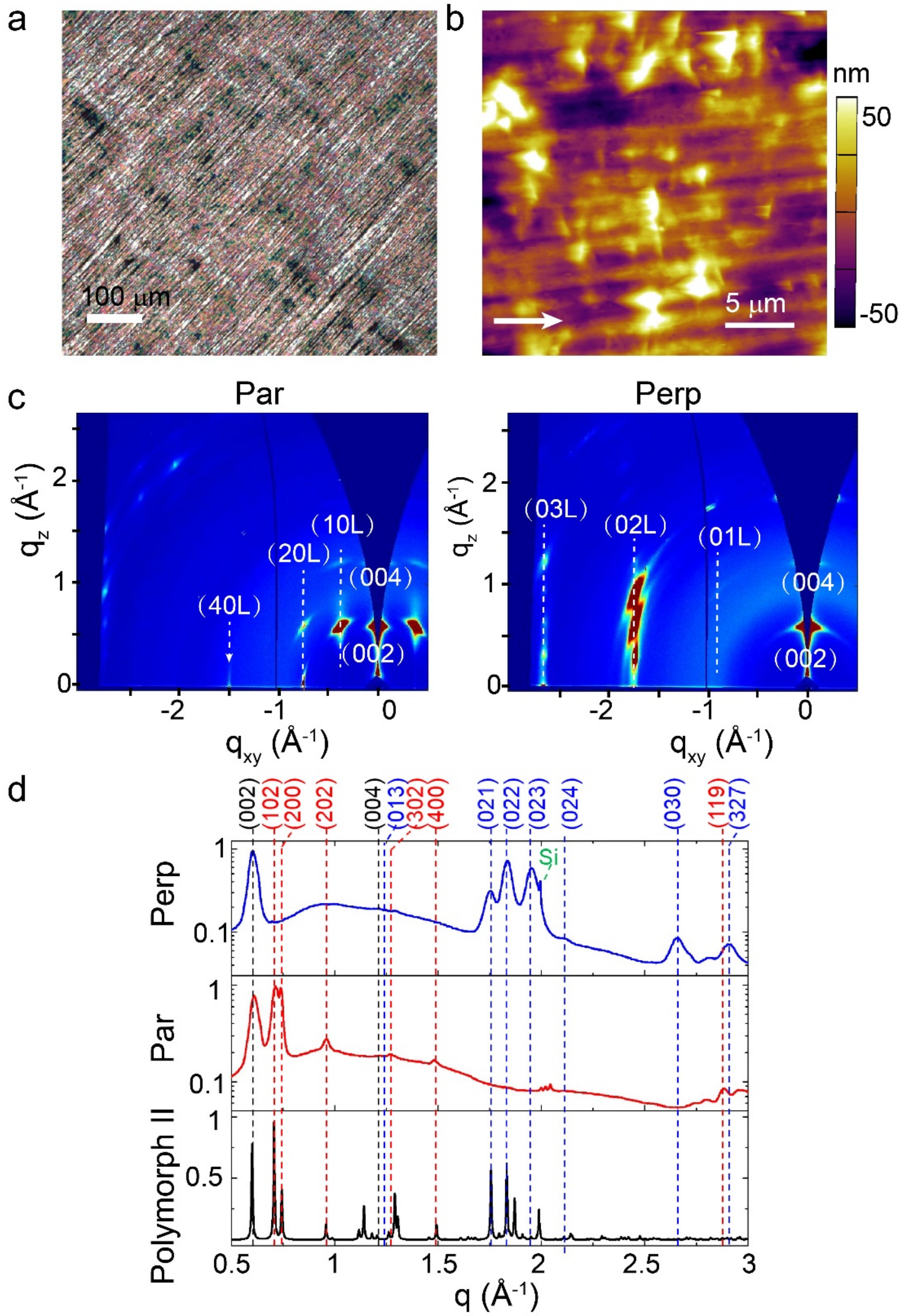

Supplementary Figure 15│ Structural characterizations of ellipticine thin films coated from THF solution on PMMA dielectric substrate. (**a**) C-POM images for ellipticine film deposited from 3 mg/ml THF solution at a coating speed of 0.05 mm/s at RT. (**b**) Tapping mode AFM height image of the coated thin film. (**c**) GIXD images of the ellipticine thin film with the incidence X-ray beam parallel and perpendicular to the coating

direction. (**d**) Comparison of 1D GIXD patterns with the simulated diffraction pattern of ellipticine from polymorph II .cif file.

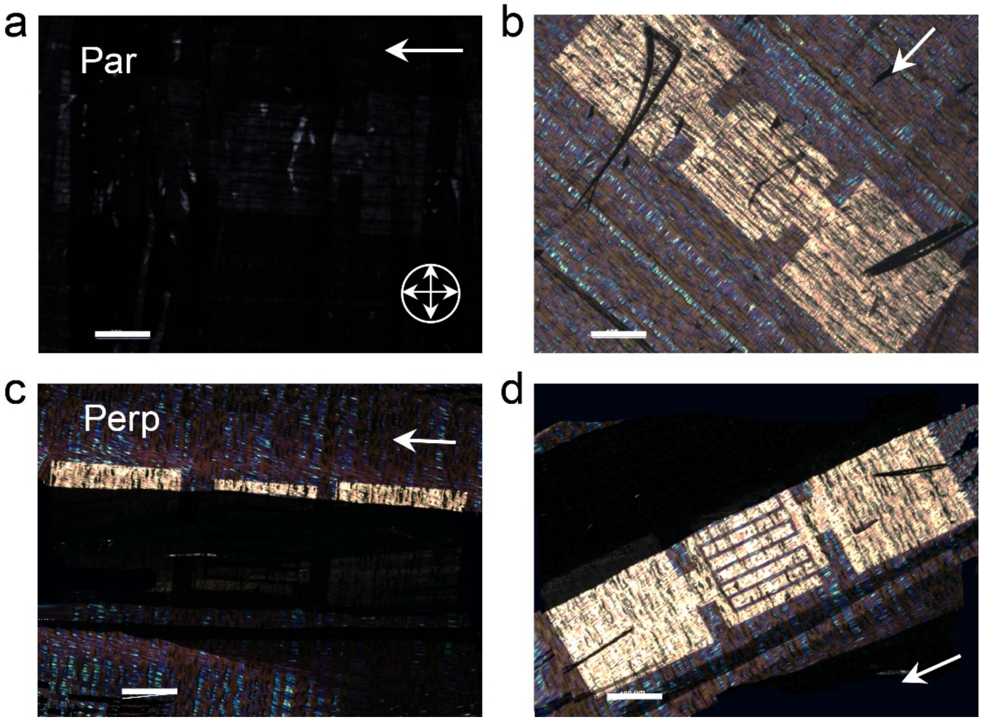

Supplementary Figure 16| OFET devices on highly aligned ellipticine domains. Cross-Polarized Optical Microscopy (C-POM) images of the Organic Field-Effect Transistor (OFET) devices with the coating direction (**a**, **b**) parallel and (**c**, **d**) perpendicular to the device channel. The white arrows denote the coating direction. The scale bars are 100 μm. The ellipticine films were solution coated from THF solution on SiO_2 substrates prepatterned with interdigitated Ti (5 nm)/Au electrodes (25 nm).

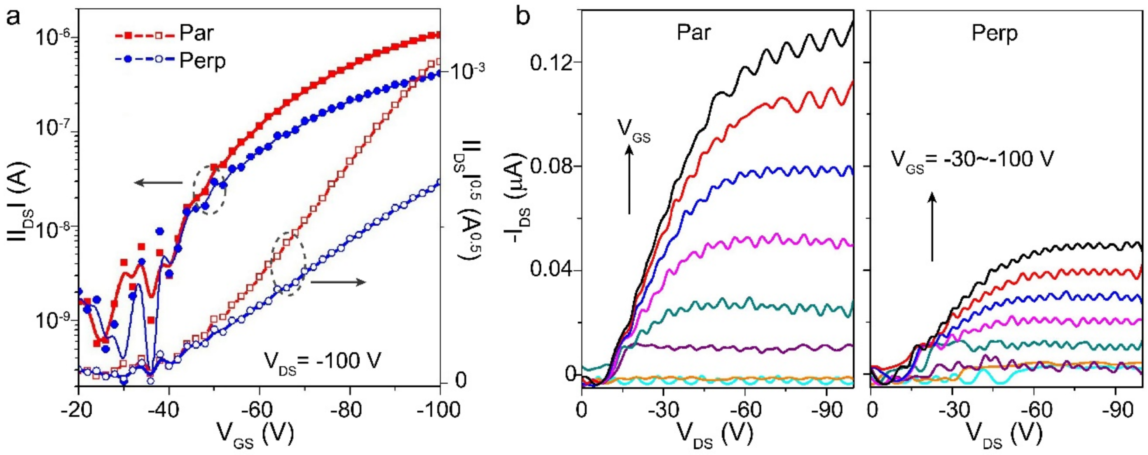

Supplementary Figure 17 | Organic Field-Effect Transistor (OFET) device performance with bottom-gate bottom-contact (BGBC) structure. (**a**) Transfer and (**b**) Output curves for ellipticine based BGBC OFET. The deep HOMO level of ellipticine causes a large charge injection barrier, and correspondingly a significant Schottky resistance. The contact resistance is reflected as a large threshold voltage in the transfer curve and 'S'-shaped in output curves. We calculated the interfacial trap state density (N_{it}) from the subthreshold swing [4,5]. N_{it} for the BGBC structure devices were estimated to be $3.5\times10^{13}\pm0.5\times10^{13}$ $eV^{-1}cm^{-2}$, which is substantially higher than the values reported in high-performance small molecule organic semiconductor OFET devices (~10^{12} $eV^{-1}cm^{-2}$)[4,6].

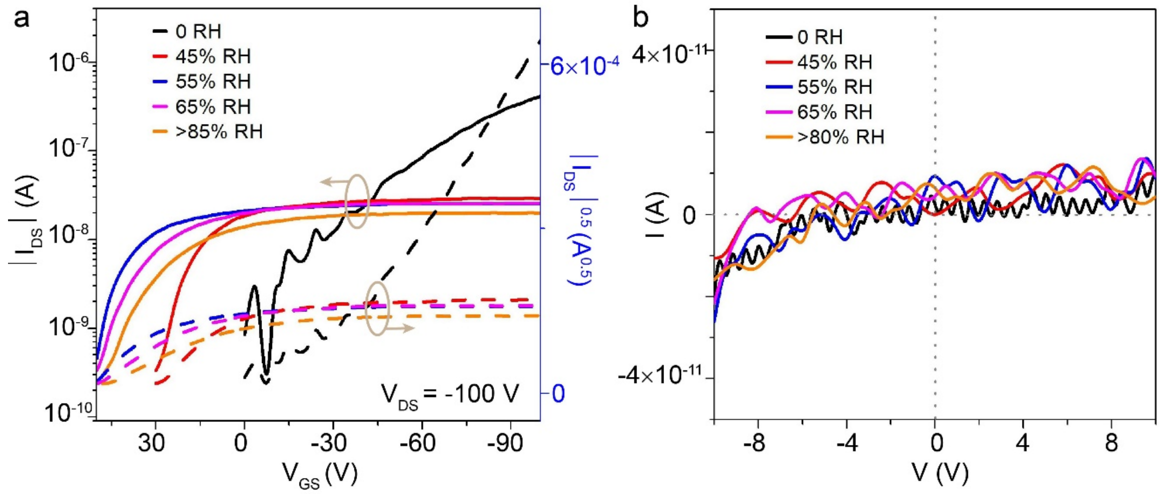

Supplementary Figure 18│ Humidity effects on ellipticine device performance. (**a**) Transfer curves under various relative humidity (RH). (**b**) I–V curves measured under different RH conditions without gate bias. The film was deposited on plasma treated Si/SiO_2 substrate from DMSO (2 mg/ml, with 10 wt% PMMA) solution. The substrates were pre-patterned with electrodes to yield bottom contact, bottom gate devices. Device channel length and width were 5 μm and 1400 μm, respectively. RH= 0 was measured in N_2 and the rest of conditions in humid air with RH controlled by water vapor. As shown in the I-V curves, the ellipticine films were non-conductive without gate bias, even in the high humidity environment, eliminating the possibility of humidity induced protonic transport. When exposed to humid air, the OFET mobility reduced and the V_{th} displayed a positive shift due to water induced traps in the conductive channel. These observations show that the carrier transport in ellipticine devices is dominated by gate bias induced holes at the ellipticine/dielectric interface.

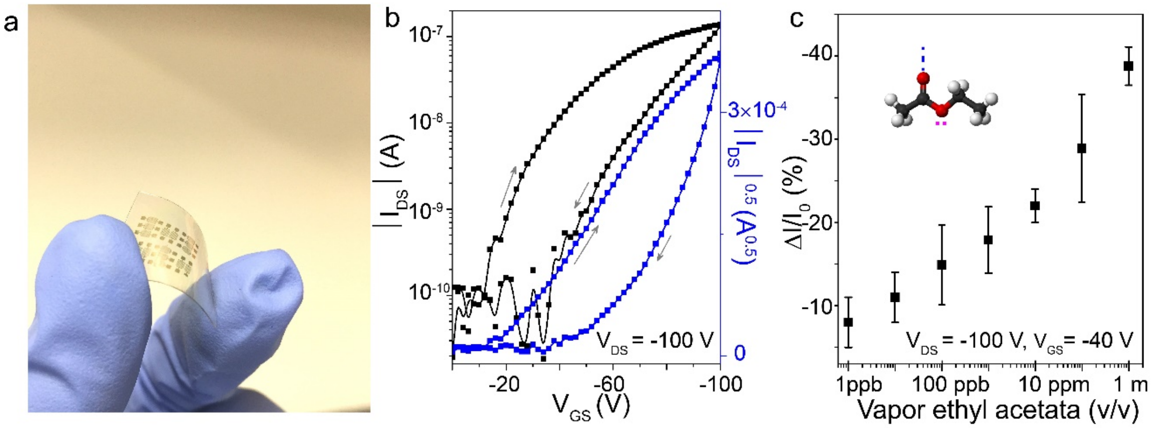

Supplementary Figure 19 | Flexible sensor chip based on printed ellipticine. (**a**) Photograph of the flexible sensor chip. The device was fabricated in the PET/ITO/CYTOP/Au/ellipticine structure. (**b**) Transfer curve of the OFET based ellipticine sensor measured in the air. (**c**) Current response to ethyl acetate vapor over a wide dynamic range.

Supplementary Tables

Material / Condition	Ellipticine	SN38	10-hydroxy-camptothecin	Amonafide
$E_{g, optical}$ in DMSO solution	2.96 eV	3.07 eV	3.05 eV	2.53 eV
$E_{g, optical}$ for Crystalline film	2.83 eV	2.95 eV	2.94 eV	2.53 eV
DFT calculated HOMO-LUMO gap*	3.80 eV	3.67 eV	3.75 eV	3.23 eV
*** See Figure 1 for details.**				

Supplementary Table 1 | Measured optical bandgap ($E_{g, optical}$) of DNA intercalators from UV-vis absorption spectra. The optical bandgaps of all compounds were estimated from the onset of the lowest energy absorption peak in the solution and solid state. These experimentally estimated optical bandgaps are qualitatively consistent with the electronic bandgaps obtained from DFT calculations for single molecules in vacuum.

**Ellipticine based crystals**	**Polymorph I**	**Polymorph II**
Crystal system	Monoclinic	Orthorhombic
Space group	P 2_1/c	Pbca
a (Å)	5.105(2)	16.8027(5)
b (Å)	15.588(6)	7.2490(3)
c (Å)	16.161(6)	20.7982(8)
α (°)	90	90
β (°)	97.03(9)	90
γ (°)	90	90
Cell volume V (Å^3)	1276.37	2533.28
Z value	4	8
R-factor (%)	5	4.91
Interplanar π−π distance (Å)	3.45	3.44
Intermolecular π−π distance (centroid-centroid) (Å)	5.10	3.64

Supplementary Table 2│ Unit cell parameters for ellipticine of polymorph I and polymorph II, obtained from single-crystal X-ray diffraction using polymorphs of ellipticine single crystals grown from DMSO solution during slow solvent evaporation[2,3]. The cif files were registered with the Cambridge Crystallographic Data Center (CCDC) as CCDC ELLIPT (polymorph I) and 1817466 (polymorph II).

**Treatments**	**Channel direction**	**Mobility Ave (max) $cm^2V^{-1}s^{-1}$**	**V_{th} Ave (min) V**	**Log (I_{on}/I_{off}) Ave (max)**

Deposited from DMSO solution	Par	4.5×10^{-5} (6.7×10^{-5})	-50 (-45)	1.8 (2.2)
	Perp	1.0×10^{-5} (1.4×10^{-5})	-55 (-49)	1.6 (2.0)
Deposited from THF solution	Par	1.5×10^{-5} (1.7×10^{-5})	-45(-43)	3.8 (4.0)
	Perp	0.8×10^{-5} (0.9×10^{-5})	-45 (-42)	3.1 (3.5)
1.5 mg/ml F_4-TCNQ doping	Par	8.9×10^{-5} (1.6×10^{-4})	-13 (-8)	2.6 (3.0)
	Perp	3.4×10^{-5} (3.9×10^{-5})	-16 (-10)	2.0 (2.4)

Supplementary Table 3│ Summary of BGBC OFET device performance. The OFETs were measured with the channel parallel and perpendicular to the coating direction. N-doped Si and 300 nm SiO_2 served as the gate electrode and the dielectric layer, respectively. Patterned on the dielectric were source-drain electrodes of 25 nm Au with 5 nm Ti adhesion layer; the channel length and width were 5 μm and 1400 μm, respectively. The ellipticine polymorph II films were deposited from DMSO or THF solution with PMMA additive on plasma treated SiO_2 substrate via meniscus-guided solution coating process (see Methods). Further surface doping was performed by spin-coating F_4-TCNQ/water solution atop the ellipticine layer (see Methods). All measurements were carried out in the glovebox.

**Treatments**	**Channel direction**	**Mobility Ave (max) $cm^2V^{-1}s^{-1}$**	**V_{th} Ave (min) V**	**Log (I_{on}/I_{off}) Ave (max)**
Deposited from DMSO solution	Par	6.4×10^{-4} (1.3×10^{-3})	-49 (-38)	2.6 (3.5)
	Perp	1.9×10^{-4} (0.4×10^{-3})	-45 (-38)	2.2 (2.5)

Supplementary Table 4│ Summary of TGBC device performance. A ~350 nm thick CYTOP dielectric layer was spin coated onto the ellipticine film (see Methods). 40 nm Cu was further deposited as the gate electrode. The devices were not doped in this case and only DMSO devices were successful due to smoother topology of the ellipticine films deposited from DMSO.

Supplementary References

1. Bredas, J. L., Calbert, J. P., da Silva Filho, D. A. & Cornil, J. Organic semiconductors: a theoretical characterization of the basic parameters governing charge transport. *Proc. Natl. Acad. Sci. U S A* **99**, 5804-5809 (2002).
2. Horstman, E. M. *et al.* Solution coating of pharmaceutical nanothin films and multilayer nanocomposites with controlled morphology and polymorphism. *ACS Appl. Mater. Interfaces* **10**, 10480-10489 (2018).
3. Courseille, C., Busetta, B. & Hospital, M. Structure cristalline et moleculaire du dimethyl-5,11-6H-pyrido[4,3-b]carbazole (ellipticine). *Acta Crystallographica Section B* **30**, 2628-2631 (1974).
4. Niazi, M. R. *et al.* Solution-printed organic semiconductor blends exhibiting transport properties on par with single crystals. *Nat. Commun.* **6**, 8598 (2015).
5. Kalb, W. L. & Batlogg, B. Calculating the trap density of states in organic field-effect transistors from experiment: A comparison of different methods. *Phys. Rev. B* **81**, 035327 (2010).
6. Zhang, F., Dai, X., Zhu, W., Chung, H. & Diao, Y. Large modulation of charge carrier mobility in doped nanoporous organic transistors. *Adv. Mater.* **29**, 1700411 (2017).
